# Localized Nanopore Fabrication in Silicon Nitride Membranes by Femtosecond Laser Exposure and Subsequent Controlled Breakdown

**DOI:** 10.1021/acsami.5c00255

**Published:** 2025-01-27

**Authors:** Chrysovalantou V. Leva, Saumey Jain, Kevin Kistermann, Kasumi Sakurai, Göran Stemme, Anna Herland, Joachim Mayer, Frank Niklaus, Shyamprasad N. Raja

**Affiliations:** 1 Division of Micro and Nanosystems (MST), School of Electrical Engineering and Computer Science (EECS), KTH Royal Institute of Technology, Stockholm SE-10044, Sweden; 2 Division of Nanobiotechnology, SciLifeLab, Department of Protein Science, School of Engineering Sciences in Chemistry, Biotechnology and Health (CBH), KTH Royal Institute of Technology, Stockholm SE-10044, Sweden; 3 Central Facility for Electron Microscopy (GFE), RWTH Aachen University, Aachen 52056, Germany; 4 AIMES, Center for Integrated Medical and Engineering Science, Department of Neuroscience, Karolinska Institute, Solna SE-17177,Sweden

**Keywords:** solid state nanopore, femtosecond-laser irradiation, laser processing, controlled breakdown, dielectric breakdown, DNA translocation, nanopore

## Abstract

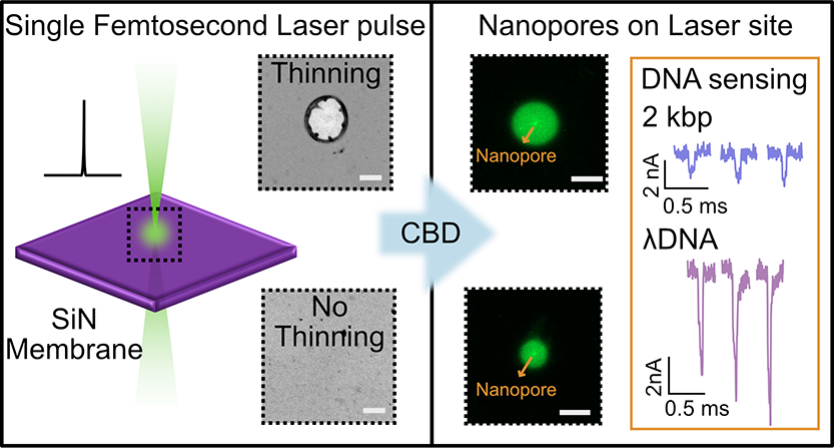

Controlled breakdown has emerged as an effective method for fabricating solid-state nanopores in thin suspended dielectric membranes for various biomolecular sensing applications. On an unpatterned membrane, the site of nanopore formation by controlled breakdown is random. Nanopore formation on a specific site on the membrane has previously been realized using local thinning of the membrane by lithographic processes or laser-assisted photothermal etching under immersion in an aqueous salt solution. However, these approaches require elaborate and expensive cleanroom-based lithography processes or involve intricate procedures using custom-made equipment. Here, we present a rapid cleanroom-free approach using single pulse femtosecond laser exposures of 50 nm thick silicon nitride membranes in air to localize the site of nanopore formation by subsequent controlled breakdown to an area less than 500 nm in diameter on the membrane. The precise positioning of the nanopores on the membrane could be produced both using laser exposure powers which caused significant thinning of the silicon nitride membrane (up to 60% of the original thickness locally), as well as at laser powers which caused no visible modification of the membrane at all. We show that nanopores made using our approach can work as single-molecule sensors by performing dsDNA translocation experiments. Due to the applicability of femtosecond laser processing to a wide range of membrane materials, we expect our approach to simplify the fabrication of localized nanopores by controlled breakdown in a variety of thin film material stacks, thereby enabling more sophisticated nanopore sensors.

## Introduction

Solid-state nanopores have gained traction as a versatile research tool for bioanalysis at single molecule resolution.^1^ Significant applications of solid-state nanopores include the detection of nucleic acids (DNA, RNA),^2^ proteins,^3,4^ and DNA–protein complexes,^5,6^ the characterization of DNA nanostructures,^7,8^ and the assembly of more complex sensors such as single protein traps.^9^ Solid-state nanopores can also be integrated with other metallic or dielectric nanostructures, which can act as additional electronic sensing channels,^10^ plasmonic antennas,^11,12^ or photonic elements^13^ to create more sophisticated sensors. Sub-100 nm diameter solid-state nanopores in suspended microfabricated membranes, most commonly made of silicon nitride, are typically fabricated using two types of pore formation techniques: (1) physical or chemical etching processes such as drilling holes using energetic electron or ion beams in vacuum,^14−16^ heavy ion irradiation followed by chemical etching of the damaged membrane (ion track-etching (ITE) technique),^17^ electron beam lithography in combination with reactive ion etching,^18^ or laser-assisted photothermal etching of silicon nitride in aqueous KCl solution;^19,20^ (2) controlled breakdown (CBD) of a membrane in an aqueous salt solution.^21^ During the CBD process, an electric field comparable to the dielectric strength of the membrane material is applied across the membrane. This drives a tunneling current across the initially insulating membrane, which seeds structural defects that accumulate over time. Some of these defects across the membrane thickness eventually merge to form a continuous conduction path through the membrane, creating an ionic conduction channel–the nanopore.^21,22^ TEM drilling, laser etching, and CBD have been used to fabricate extremely small nanopores (sub-5 nm), which have been used to demonstrate various novel single biomolecule sensing applications.^16,20,22−24^ The location of nanopore formation on a membrane when using the physical and chemical etching processes such as TEM and laser drilling can be specified, whereas, using CBD, the nanopore forms at a random location on an unpatterned membrane.^25^ For certain applications such as plasmonic nanopores^26^ or electrode-integrated nanopore devices,^27,28^ which require more precise pore positioning on a membrane, local thinning of a membrane using cleanroom-based microfabrication processes or ion-milling has been used to define the site of the nanopore formation by CBD.^29−31^ Applying a continuous focused laser spot on a membrane during the CBD process itself, requiring CBD to be carried out with the membrane mounted in an optical microscope with a laser source, has also been used to localize the site of nanopore formation.^11,32^ Two additional methods which use CBD for localized nanopore formation on silicon nitride membranes are performed with one side of the membrane immersed in electrolyte, and the other side in air. The voltage bias to induce breakdown is applied locally on the membrane from the side in air either using a conductive tip in an atomic force microscope,^33^ or by microfabricated on-chip electrodes.^34^

Of all the nanopore fabrication techniques, CBD requires the simplest setup and can be implemented using a standard DC voltage source and ammeter.^22,35^ Furthermore, laser-assisted photothermal etching and CBD are carried out in aqueous salt solutions and thereby produce readily wet nanopores,^19,24,36^ and nanopore fabrication using both techniques has been automated using custom-made experimental setups.^20,22,37^

In recent years, pulsed-laser micromachining processes have emerged as capable nanostructuring tools for biomedical, microelectronics, and photonics applications.^38−40^ Direct lithography-free machining of lines and holes down to ∼50 nm critical dimensions in supported thin films and 2D materials have been realized.^41−46^ More recently, attention has turned to studying the machining capabilities of picosecond and femtosecond pulsed lasers on suspended sub-100 nm thick films.^47−50^ The main focus of these studies on suspended films has been to determine optimal conditions to achieve complete material removal in a part or the whole of the laser-exposed area and to create novel patterned and suspended nanostructures.^47,48,50^ Although the possibility of locally thinning a suspended membrane using femtosecond laser processing has emerged from these studies,^50,51^ systematic investigations of the achievable level of controlled material removal along the *z*-axis in nanometric suspended membranes are lacking. It is unclear whether pulsed laser micromachining can be used for controlled localized thinning in the nanometer scale and whether internal structural defects can be seeded in a material even below the ablation threshold. Such information would be critical to further developing pulsed laser micromachining capabilities, which can substitute for cleanroom-based processing or focused ion-beam milling of nanostructures.

**Figure 1:**
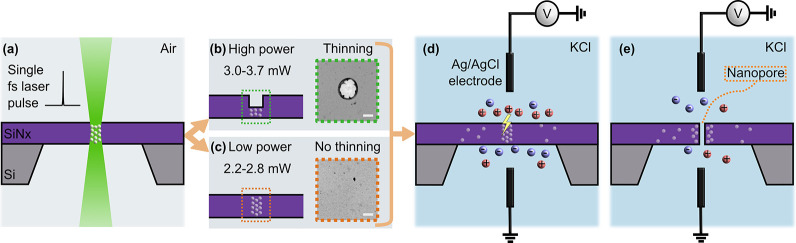
Schematic of our femtosecond laser enabled approach for localized nanopore formation by controlled breakdown (CBD). (a) Single pulse femtosecond (fs) laser exposure (λ= 520 nm) of 50 nm intact silicon nitride membrane created an initial local modification of the freestanding film. The process was performed in air. When visible modification of the membrane was observed, the modified area is 150–500 nm in diameter. We observed two regimes of damage: (b) silicon nitride thinning at high powers and (c) no visible thinning at low powers. Bright-field TEM imaging helps establish these regimes. The scale bar is 200 nm. (d) CBD of the laser-exposed membranes in a 1 M KCl solution was used to perform nanopore fabrication. Membranes from both regimes (thinned and not thinned) were investigated. (e) Nanopore formation by CBD was confirmed by electrical characterization afterward. Note that the diameter of the nanopore fabricated using our two-step process formed (5–30 nm) is typically 10–50 times smaller than the laser-modified area.

Here, we report an approach using localized single-pulse femtosecond laser exposure of a thin silicon nitride membrane to define the spatial position of nanopore formation by controlled breakdown. In our two-step process, laser exposure of the membrane is first performed in air, following which nanopores are fabricated by CBD in salt solutions (Figure 1a). Using a combination of TEM analysis, fluorescent microscopy, and the CBD process itself, we identify two broad regimes of damage caused to the membrane by a single laser pulse depending on the nominal power of laser exposure: one regime above the ablation threshold which causes localized thinning, and another below the ablation threshold (Figure 1b, c) which causes internal material damage but no visible change to the membrane. From our investigation, CBD emerges as a sensitive indirect technique to probe defect generation in thin suspended films. We show that nanopores of diameter down to ∼ 5 nm localized to an area less than 500 nm in diameter can be made using our two-step approach, i.e., single pulse laser exposure in the air followed by CBD in liquid (Figure 1a, d, e).We demonstrate the validity of this approach to creating nanopore sensors by performing double-stranded DNA translocations using fabricated nanopores.

## Results and Discussion

To investigate localized laser-induced damage of a suspended membrane and to demonstrate its utility in defining the location of nanopore formation by CBD, we used 50 nm thick silicon-rich silicon nitride membranes fabricated using standard microfabrication processes as the test substrate. We first exposed several silicon nitride membranes to single femtosecond laser pulses at different laser powers in air. Then, we performed the CBD process in 1 M KCl to determine the dependence of the pore formation processes on the laser power. The power of the laser exposure is the average power measured after the objective. The conversion from the power values to the laser pulse energy values is described in the Methods section. The diameter of the focused laser spot was ∼1 μm in all the experiments. In the CBD process, a voltage bias large enough to cause gradual dielectric breakdown of the membrane material is applied across the membrane (30 V in our case). The time it takes to form a nanopore, measured as the duration from the instant the voltage bias is applied until nanopore formation is detected as an abrupt increase in current, called time-to-breakdown (T_BD_), is a characteristic of the CBD process. If all conditions are identical, T_BD_ typically follows a certain Weibull distribution since dielectric breakdown is a stochastic process.^52^ Any significant prior physical damage to the membrane (e.g., by laser exposure) would be expected to have a measurable statistical impact on T_BD_. Therefore, we used T_BD_ as the first metric to track whether laser exposure at a certain power had a measurable impact on the nanopore fabrication process.

**Figure 2:**
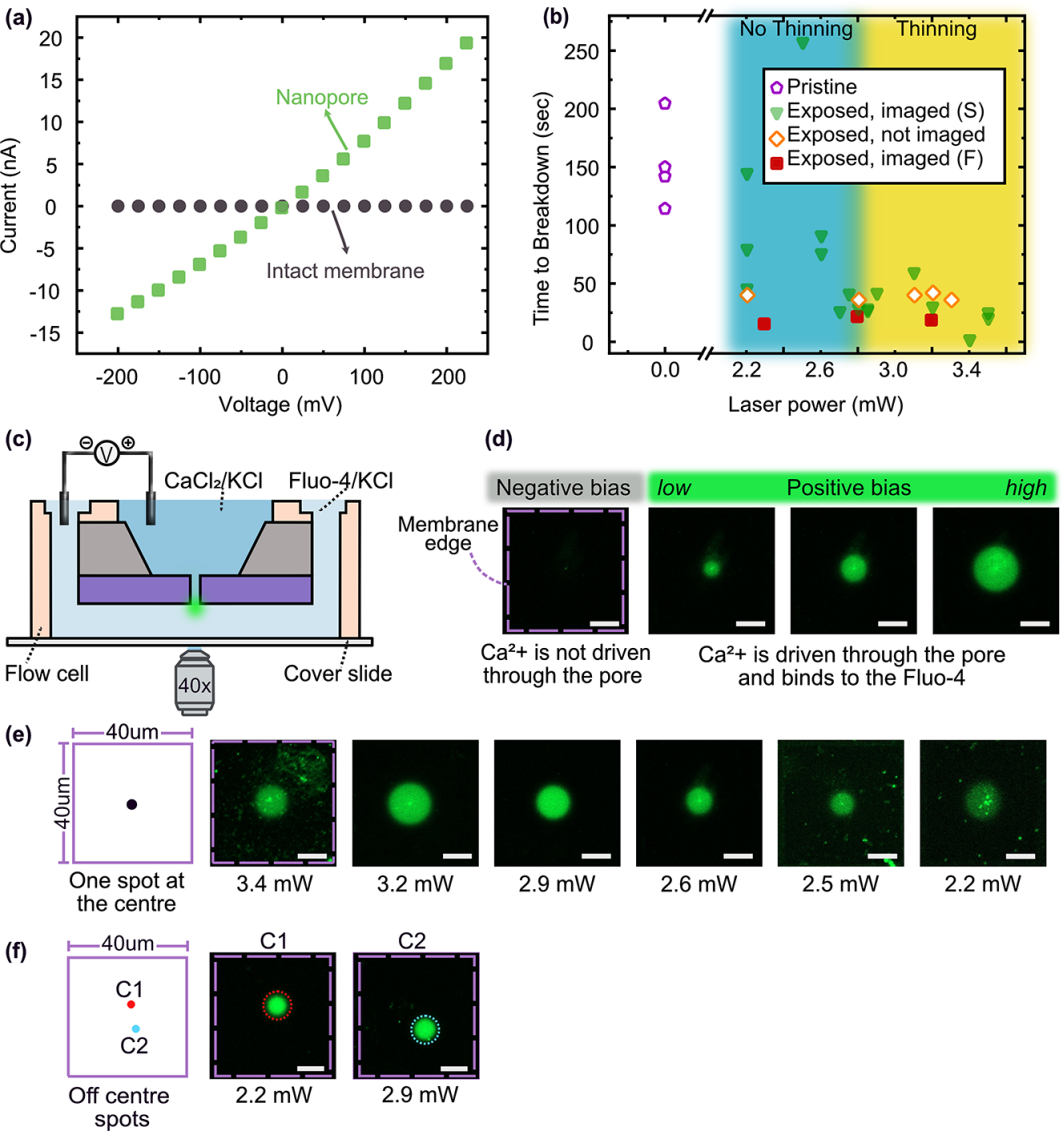
Electrical characterization and fluorescent optical visualization of localized nanopore formation by CBD after laser exposure. (a) *I*–*V* characteristics of a typical laser-exposed membrane before and after the nanopore formation by CBD. (b) Comparison of time-to-breakdown (T_BD_) for 25 laser-exposed and 4 unexposed membranes. Nanopores that formed at the desired location on a membrane are marked with an “S″ and those which did not with an “F″. Overall T_BD_ decreased as the laser power increased. The two regimes of damage to the membrane as a function of laser power (“no thinning” and “thinning”) are also indicated. (c) Schematic of fluorescent optical visualization of nanopores in a confocal microscope. A calcium ion (Ca^2+^) sensitive dye (Fluo-4) was used to locate the position of a nanopore on a membrane by driving Ca^2+^ electrically through the nanopore (d) to produce a circular fluorescent spot whose area increases with increasing voltage bias of positive polarity (2–4 V). At negative bias (−500 mV), Ca^2+^ is not driven through the nanopore, so no signal is observed. The nanopore is located at the center of the observed fluorescent spot. The purple dashed line represents the edge of the suspended membrane. (e) Image series showing the successful localization of nanopore formation to the site where laser exposure was performed (center of the square membrane in each case) for a range of nominal laser powers from 3.4 to 2.2 mW. The purple box represents the membrane’s edges. (f) In two instances (C1, C2), laser exposure was deliberately performed off-center, which still produced nanopores at the desired locations. The scale bar is 10 μm for all panels in (d–f).

To perform nanopore fabrication by CBD after single pulse femtosecond laser exposure, each membrane chip was mounted in a flow cell to create separate fluidic reservoirs for the two sides of the membrane following well-known procedures (see Methods). After wetting both sides of the membrane in 1 M KCl, we performed current–voltage (I–V) sweeps to verify that only a leakage current was measured, and that the membrane did not already have a through-hole at this stage. After nanopore fabrication using the CBD process, an I–V sweep showed an ohmic response, indicating the successful formation of a nanopore (Figure 2a).

We performed CBD on 25 membranes exposed at various laser powers ranging from 2.2–3.5 mW and eight pristine membranes that were not exposed to the laser before the CBD process as controls (Figure 2b). Overall, we observed that the time-to-breakdown decreased as the laser power increased, which indicates that in the selected range of laser powers, there is an increasing degree of laser-induced damage to the membranes. Note that in Figure 2b, we only include those pristine membranes in which nanopore formation occurred within 1000 s, while four more pristine membranes were evaluated in which no nanopore formation was observed while applying the bias voltage for 1000 s. In our experiments, we found that the lowest laser power that resulted in a measurable decrease in the breakdown time of the CBD process was 2.2 mW (Figure 2b). We see that the upper limit of the breakdown times for the samples that were exposed to the 2.2 mW laser pulse overlaps with the lower limit of the breakdown times of the pristine membranes which yielded nanopores in the CBD process.

To confirm that the nanopores indeed form at the location determined by the laser exposure on our membranes, we used an optical visualization technique previously used to locate the site of nanopore formation.^29^ In this technique, a fluorescent dye (Fluo-4) whose fluorescence intensity increases by several orders of magnitude when it binds with calcium ions (Ca^2+^) is used to locate the position of a nanopore. By filling the fluid chambers on either side of a membrane containing a nanopore with a buffer containing Fluo-4 on one side and calcium ions on the other side, a circular area of fluorescent emission centered around a nanopore can be observed when an applied voltage bias drives calcium ions through the nanopore (Figure 2c). A hallmark of this technique is that the size of the circular area increases with increasing voltage bias of the correct polarity (positive in our case), as this drives an increasing amount of calcium ions through the nanopore to the other side. A dark background with no emission above the background level is produced when a voltage bias of the opposite polarity is applied (Figure 2d). Using this approach, we imaged a total of 20 membranes with nanopores formed by CBD that were previously exposed to a single laser pulse at various laser powers spanning the range of 2.2–3.5 mW. We found that in 17 instances, the nanopore was located at the location of laser exposure (Figure 2b). In the three other instances, the nanopore was formed elsewhere on the membrane. It is worth noting that the breakdown times for these three cases were among the lowest that we observed, indicating that pre-existing defects at other locations on the membrane might have rendered whatever damage was caused by laser exposure inconsequential in these cases (2 of 3 nanopores formed at the edge of the membrane; SI-Figure S1). Figure 2e shows a series of samples exposed to a single laser pulse of different powers at the center of the membrane. We achieved successful nanopore localization down to a laser power of 2.2 mW. Even though nanopore formation is localized to the spot of laser exposure, without direct TEM imaging we cannot conclusively determine whether one or multiple closely spaced nanopores were formed after CBD. However, we know from various studies in literature that it is possible to tune the controlled breakdown process to the extent that a single nanopore is the most likely outcome.^22^ This is done by (1) stopping the application of high voltage bias within ∼1s after the detection of the first nanopore formation and (2) not applying more than ∼one-third of the electric field required for breakdown afterward, e.g. when characterizing the pore or while performing nanopore sensing. In our implementation of CBD, the voltage termination delay was ∼1–10s. The nanopores formed in this way were typically of effective single nanopore diameter equivalent >15 nm. We interpreted this to have been due to pore expansion after formation, during the voltage termination delay, as no additional current spikes indicating new pore formation were observed in the signal after the initial spike. This is because as soon as the first nanopore forms the electric field in its vicinity drops drastically, making it highly unlikely that a second nanopore forms.^53^ We achieve faster voltage termination (<300 ms) by using a pulsed CBD process as described later. In Figure 2e, several tiny bright spots can be observed at several locations of the membranes where no laser exposure was performed (e.g., the panels exposed at 3.4, 2.5, and 2.2 mW). We found that unlike nanopores these bright spots do not change in shape or fluorescent emission intensity in response to the applied voltage bias (SI-Figure.S2 (a), (b)). We attribute these spots to debris on the membrane which likely originate from the repackaging process of the chip after CBD, when the chip was transferred into another flow cell that was suitable for the fluorescent imaging procedure (see Methods).

To evaluate the capabilities of the technique to localize the nanopore at different locations of the membrane, we changed the position of laser exposure and successfully fabricated nanopores at off-center locations of the membrane. Two such examples are shown in Figure 2f using laser powers of 2.2 and 2.9 mW, respectively. We also present preliminary results which indicate that it is possible to form multiple nanopores on predefined spots on a membrane by laser exposure of an array of 3 × 3 spots prior to performing the CBD process (SI- Note 1 and Figure S3).

**Figure 3:**
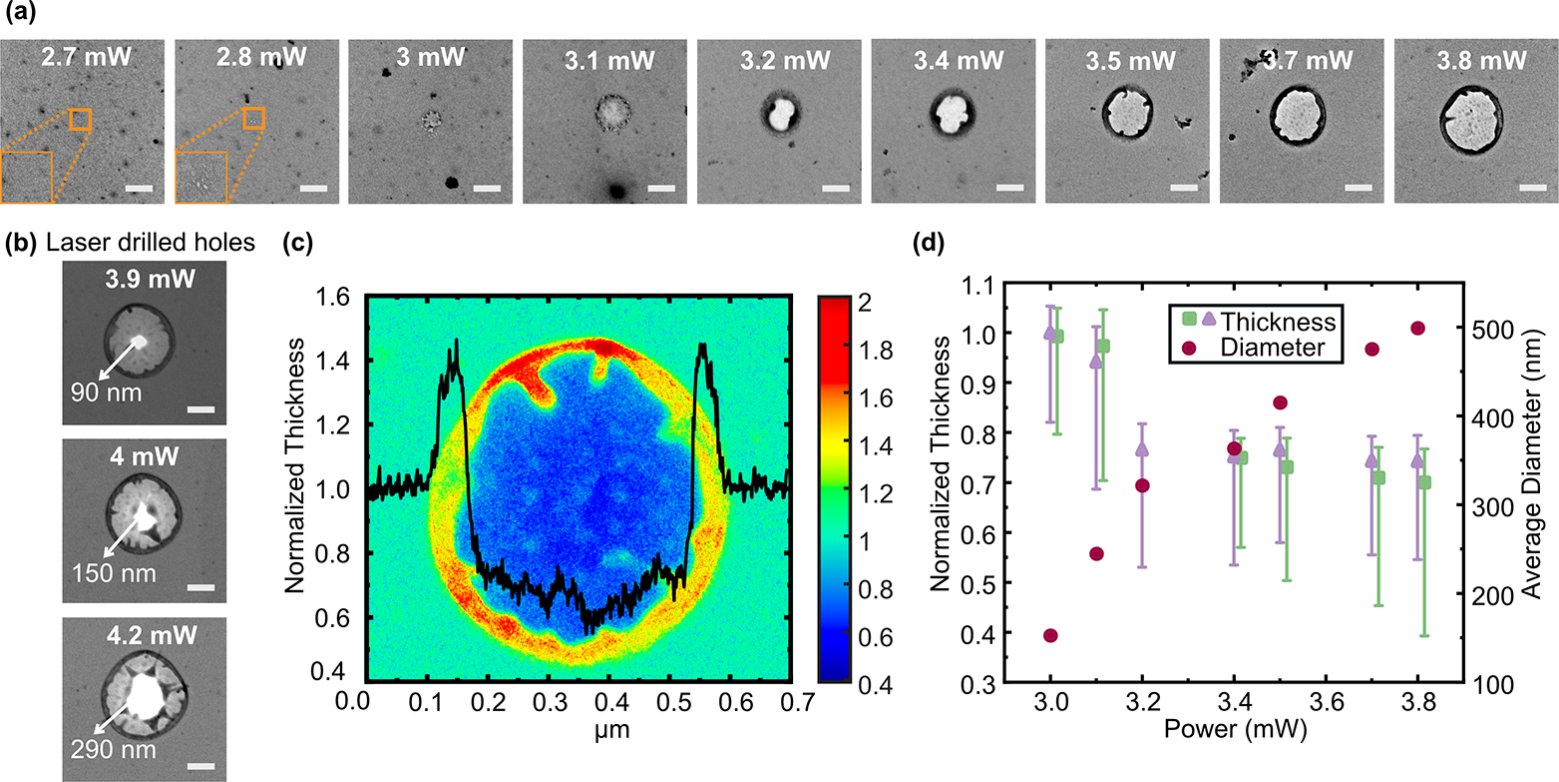
TEM analysis of the morphology and topography of laser-exposed silicon nitride membranes. (a) Bright-field TEM (BF-TEM) images of membranes exposed with a single femtosecond pulse at different laser powers in the range 2.7–3.8 mW. At 2.7 mW, there was no visible change to the membrane. From 2.8 mW onward the diameter of the laser-modified area increases with increasing power. Insets at 2.7 and 2.8 mW show magnified views of the central area. (b) At higher laser exposure power than 3.8 mW, BF-TEM images show laser-drilled holes. The equivalent circular diameter of three such holes drilled at laser powers 3.9, 4, and 4.2 mW are also shown. (c) A normalized thickness map and line profile of a sample exposed at 3.8 mW were obtained using EFTEM imaging. From the thickness map, we observe that the laser-modified area consists of a central thinned region (blue) enclosed by a thick rim (yellow). The unthinned area of the membrane surrounding the laser-modified area is also shown (green). The line profile is extracted along a central horizontal line (700 nm) running across the entire width of the map. The line profile was created by averaging 10 pixels along the vertical axis. The color bar represents the normalized thickness scale. (d) Diameter of the laser-modified area and the normalized thickness of the thinned region of membranes (inside the red rim in part d) after laser exposure at various powers obtained from EFTEM analysis. The markers (△, ○,□) indicate the average thickness; the minimum thickness is shown as the negative error bar, and the uncertainty in thickness estimation as the positive error bar. The measurements shown here originate from different well-separated (>5 μm; diffraction-limited laser spot size was <1 μm) locations on the same membrane where a series of laser exposures at different powers were carried out to perform this analysis. The scale bar for parts a, b is 200 nm.

To elucidate what kind of damage occurs to the suspended silicon nitride membranes during exposure to a laser pulse at various powers, we used BF-TEM (Bright-Field Transmission Electron Microscopy) and EFTEM (Energy-Filtered Transmission Electron Microscopy) to study the morphology of the damaged area of the silicon nitride membranes. The BF-TEM images (Figure 3a, b) showed that the visible ablation threshold occurs at a laser power of around 2.7–2.8 mW: below this threshold, we observed no visible change to the silicon nitride membranes; above this threshold, there was a visible change to the membrane at the location of laser exposure (Figure 3a). The area of visible modification increased with increasing laser power until a nanopore was directly drilled at the center of the modified area, which occurred at a laser power of 3.9 mW, resulting in a hole with an equivalent circular diameter of approximately 90 nm (Figure 3b). Using EFTEM imaging we investigated the topography of the laser ablated region of the silicon nitride membranes for laser powers between 3.0–3.8 mW. The lowest power at which one contiguous laser-modified area was observed is 3 mW, while at 2.8 mW, a disconnected cluster of nanoscopic laser-modified areas was observed (Figure 3a; SI-Figure S3). The typical topography of a laser-damaged area for powers greater than 3 mW was a thinned central region enclosed by a narrow raised rim surrounded by the unthinned membrane area (Figure 3c). From the EFTEM images we computed the average and the minimum thickness of the central thinned area (i.e., the area inside the rim; Figure 3c). The average thickness is an overall measure of material removal in the thinned area and is relevant to understand the efficacy of localized thinning using a single laser pulse at a specific power. The minimum thickness, on the other hand, is a single pixel measurement within the overall thinned area and is important for the CBD process, as it is probable that nanopores have the highest likelihood of forming first at the thinnest part of the membrane since this is where the electric field would be the highest. In the following, we discuss the average and minimum thickness in terms of percentage values, where 100% is the pristine membrane thickness (∼50 nm in our case). We observed significant thinning only from a laser power of 3.2 mW. The average thickness decreases almost imperceptibly from ∼76% at 3.2 mW to 70–74% at 3.8 mW (Figure 3d). The minimum thickness, on the other hand, shows a strong dependence on the laser power. It is already significantly different from the pristine thickness at 3 mW. The minimum thickness decreases from ∼80–82% at 3 mW to 39–55% at 3.8 mW (Figure 3d). Note that the ranges indicate measurements from two independent areas on the same membrane separated by several micrometers and exposed at the same laser power. From the average thickness measurements, we infer that material removal along the *z*-axis is not a gradually increasing function of the laser power. Instead, it increased in a ∼20% step between 3.1–3.2 mW (where significant thinning was first observed) and a ∼70% step between 3.8–3.9 mW (where the laser drilled a through hole). Therefore, finer control of power around these two laser power thresholds might be required to achieve greater control over thickness reduction using a single femtosecond laser pulse over the complete range of membrane thickness from 0–100%. To achieve laser powers after the objective in the range of 2.2–3.8 mW, we set the laser emission power to 170–230 mW and used a Neutral Density (ND) filter with an optical density of 1.0 to attenuate the beam, and the measured power after the objective varied by ±0.02 mW. Finer control of the laser power delivered might be possible to achieved in future in two ways: (1) using a higher set power and a stronger ND filter, it might be possible to decrease laser power deviations after the objective to a 0.01 mW level, e.g. by using 3 W laser emission power and Optical Density (OD) = 10 to achieve ∼ 3 mW after the objective; and (2) by using a few rather than a single pulse. EFTEM maps for all investigated laser powers is presented in SI (Figure S5). One alternative strategy to EFTEM for calibrating the localized laser thinning of membranes could be atomic force microscopy. In addition, a fast but indirect measurement of laser thinning using a local measurement of optical transmittance might be possible to use, to estimate the membrane thickness after the laser thinning process once an initial calibration using EFTEM or AFM has first been performed.^49^ Since surface roughness could impact nanopore function, we used EFTEM derived thickness profiles to estimate rms roughness of a laser thinned membrane area. We used a 10-pixel wide thickness profile extracted from the sample exposed at 3.8 mW (Figure 3c). We first fit a second-order polynomial to “flatten” the profile and computed the rms roughness to be ∼0.9 nm. In the adjacent unexposed membrane area, the rms roughness (without any profile flattening) was computed to be ∼ 0.7 nm. These estimates indicate that within the thinned area, the increase of the local surface roughness after laser thinning is minimal.

From the TEM analysis, we identified the lowest laser power at which any visible modification of the membrane occurred to be ∼2.8 mW. However, using fluorescent optical visualization, we found that 6 (out of 7) samples exposed at laser powers between 2.2–2.6 mW produced nanopores by CBD at the site of laser exposure (Figure 2b, c). This indicates that despite the absence of visible laser ablation, structural defects were seeded in the laser-exposed area of the silicon nitride membranes and that these defects were significant enough to localize nanopore formation by CBD. From these results, we also discovered that the CBD process is a sensitive indicator of the fact that a certain nanometric volume has been modified by laser exposure– something that was not possible to do using BF-TEM or EFTEM imaging.

**Figure 4:**
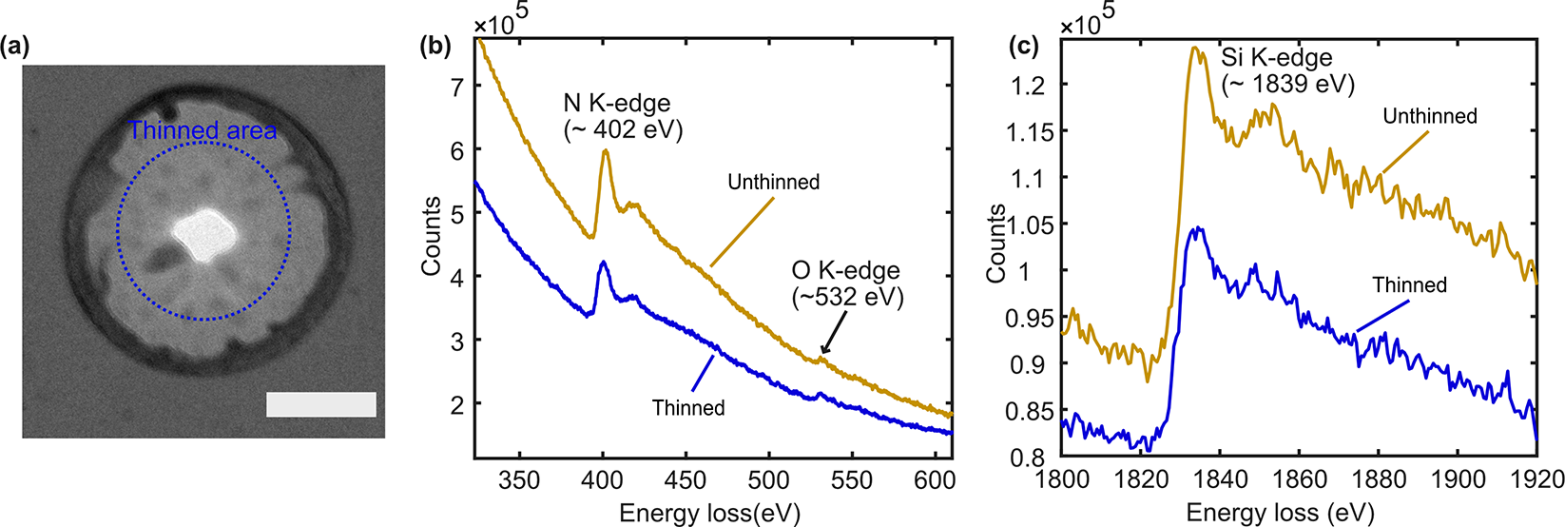
Chemical composition (Si, N, and O) of laser-thinned and unthinned areas were compared using electron energy loss spectroscopy (EELS). (a) BF-TEM image indicating the 300 nm diameter circular area from which the EELS spectra representing a laser-thinned region were collected. The EELS spectra for an unthinned region were collected from an adjacent membrane area outside the dark rim. (b) The N K, O K, and (c) Si K edges are all observed in both the thinned and the unthinned regions. The absolute counts are lower in the thinned region than in the unthinned region, and this can be attributed to the smaller sample volume in the thinned region. Scale bar for part a is 200 nm.

Furthermore, we used EELS (Electron Energy Loss Spectroscopy) to determine whether any significant change in chemical composition had occurred in the thinned membrane areas due to laser exposure, particularly to the nitrogen and oxygen content of the silicon nitride membrane. To do this, we acquired EELS point spectra using a membrane exposed at a laser power of 3.9 mW, which had a laser-drilled nanopore at the center, a thinned region around the nanopore, surrounded by a thicker rim, and an unthinned membrane area all around that (Figure 4a). The point spectra including the N K edge (∼ 402 eV) and O K edge (∼532 eV) of the thinned and unthinned regions showed that both regions contain a small amount of oxygen, likely originating from surface oxidation (Figure 4b). Both regions also showed significant nitrogen peaks, as expected from a silicon nitride membrane. The thinned area showed lower overall signal intensity compared to the unthinned area, which can be explained by the overall sample volume being smaller here. Furthermore, the Si K edge (∼ 1839 eV) is prominent in the point spectra from both thinned and unthinned regions, indicating significant Si content in these volumes (Figure 4c). Overall, the EELS analysis indicated that the laser exposure did not significantly alter the chemical composition of the thinned membrane area.

**Figure 5:**
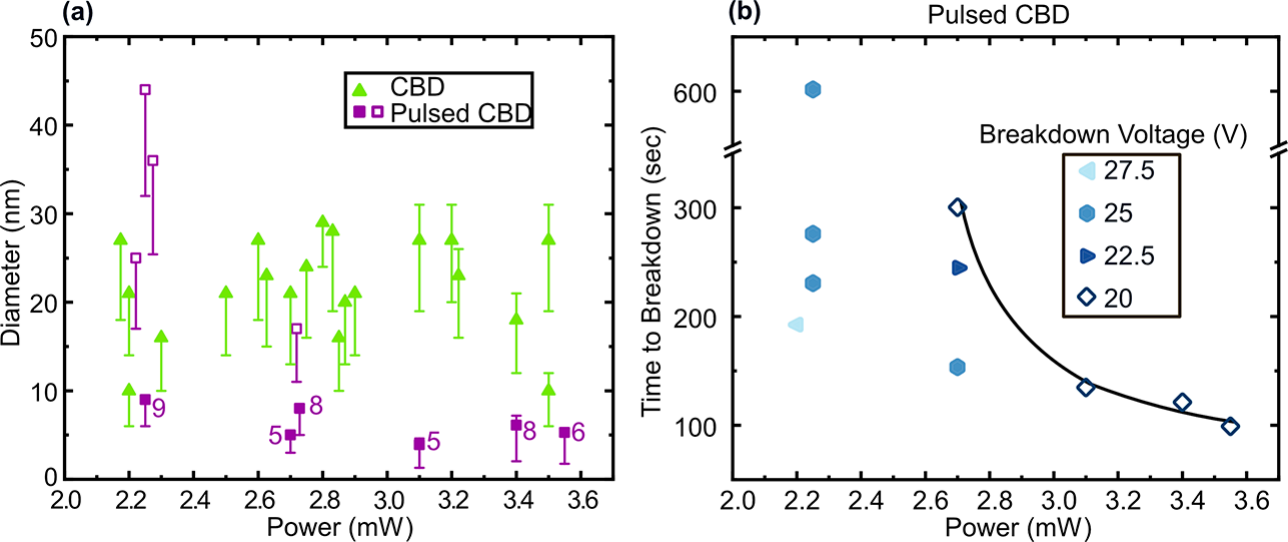
Diameter and time to breakdown of nanopores fabricated by pulsed CBD after laser exposure at various powers. (a) Diameter of nanopores fabricated by CBD (*N* = 20) and pulsed CBD (*N* = 10) after laser exposures at various powers are shown. The nominal diameters, represented by the markers, are calculated assuming that the nanopore length (L) is equal to the minimum thickness of the membrane (*t*_min_) after laser thinning, as estimated from EFTEM imaging. The error bars represent the maximum and minimum estimates of the nanopore diameter as described in the text. Note that for laser powers <3 mW the upper error bar is not shown because no measurable thinning was observed. Sub-10 nm diameter nanopores were readily fabricated using pulsed CBD in membranes exposed at higher powers, where laser exposure produced significant membrane thinning. (b) Time to breakdown of the pulsed CBD process versus the laser power that was used to expose the membranes prior to the pulsed CBD process. The different markers represent the voltage at which the nanopore formation occurred. Membranes exposed at low laser powers when no significant membrane thinning occurs (<3 mW), required higher voltages to break down compared to membranes exposed to higher laser powers associated with significant thinning of the membrane. The time to breakdown decreases with increasing laser power for four samples (connected by a solid line as a guide to the eye), where nanopore formation by pulsed CBD occurred at the lowest tested voltage bias of 20 V.

So far, we have shown how using CBD has made it possible to fabricate nanopores at the location of laser exposure. The diameter of the resulting nanopores was in the range of ∼ 10–30 nm (Figure 5a) and producing nanopores with smaller diameters was mainly limited by the high voltage bias (30 V) and the slow feedback loop used to turn off the voltage bias as soon as nanopore formation was detected. Nanopore diameters in this discussion refer to the values computed using the standard ionic conductance model for a cylindrical nanopore,^54^ which relates the measured nanopore conductance (G) to the solution conductivity (*s*), nanopore diameter (*d*) and nanopore length (L) as, . As discussed later in the text, the nanopore length is not precisely known after the breakdown process, which is a major cause of uncertainty in our nanopore diameter estimates. In the following we discuss our estimates of the nominal nanopore diameter (used as the representative value in all discussions) assuming that L = *t*_min_, where *t*_min_ represents the minimum thickness of the membrane after laser thinning as obtained from EFTEM analysis. At powers where no laser thinning was observed, L is taken as the pristine membrane thickness (t) to compute the nominal diameter (since *t*_min_ = t). The upper limit of the nanopore diameter for samples exposed at laser powers which produced thinning that was visible by EFTEM (>3 mW) was calculated assuming that L equals the average thickness of the laser-thinned region of the membrane. The lower limit of the nanopore diameter in all cases was calculated assuming L = *t*_min_/ 3 to account for tapered pore profiles after CBD. These upper and lower diameter limits are shown as upper and lower error bars in Figure 5a (SI – Table S1). The uncertainty in the nanopore diameter estimates varies between 17% and 44% of the nominal value and decreases with decreasing diameter of the resulting nanopores. To find out whether we could form localized sub-10 nm diameter nanopores using our laser-exposed samples, we used a pulsed CBD technique. Pulsed CBD processes, using an alternating series of high and low voltage pulses rather than a continuous application of high voltage, have previously been reported as a way to fabricate nanopores with smaller diameters.^55^ We performed pulsed CBD on ten samples, each exposed at a single spot with various laser powers in the range from 2.2 to 3.6 mW. We found that, indeed, we were able to fabricate sub-10 nm diameter nanopores in six out of ten instances (4.6 to 9.0 nm diameter). Eight of these nanopores show little or no asymmetry in the I–V characteristics after pulsed CBD, while two show significant asymmetry (SI-Figure S6). In these two cases we used the higher of the two conductances for nanopore diameter calculation to not underestimate the nanopore diameter. The largest nanopores were produced at the low end of laser power, where no visible damage to the membrane was seen in the TEM analysis of similar samples. The smaller nanopores were all produced at laser powers which produced visible membrane damage and significant local membrane thinning. Note that in these experiments, we started by applying 1000 pulses at 20 V, and if nanopore formation did not occur in that time, we increased the bias by 2.5 V and applied 1000 more pulses, and so on, until nanopore formation was observed (see Methods for details of our implementation of pulsed CBD). The total breakdown time and voltage at which breakdown occurred also provided an indirect measure of the magnitude and variability of the damage produced by the laser exposure (Figure 5b). For laser powers between 2.7–3.6 mW, we observed the shortest breakdown times and successful nanopore formation at the lowest voltage tested (20 V). Four of these samples were exposed to different laser powers (2.6–3.4 mW) and resulted in pore formation at 20 V, even showing a correlation between laser power and breakdown time. At a laser power of 2.7 mW, all three evaluated samples required three different voltage levels to breakdown, showing that in this transition zone between visible ablation and no ablation, there is greater variability in the magnitude of laser-induced damage compared to when higher laser powers were used. Overall, we found that sub-10 nm nanopores can be fabricated using pulsed-CBD at high yield (5 out of 6) when membranes are exposed to laser powers which produces visible damage to the membranes. This is consistent with the expectation that smaller nanopores are easier to form on thinner membranes or on membranes where the breakdown occurs at a lower voltage so that nanopore expansion after formation and before the voltage bias is turned off is not as pronounced.^29,55^ After nanopore fabrication the samples were stored in 1:1 ethanol:DIW solution until they were used for sensing experiments. By tracking the change in nominal diameter of five samples after storage for up to 4 days, we observed nanopore expansion of 1–1.5 nm/day in two cases, and nanopore shrinkage of 0.3–2 nm/day in three cases (SI – Table S2). From these limited statistics we see no obvious tendency for the nanopores to either shrink or expand during storage.

**Figure 6:**
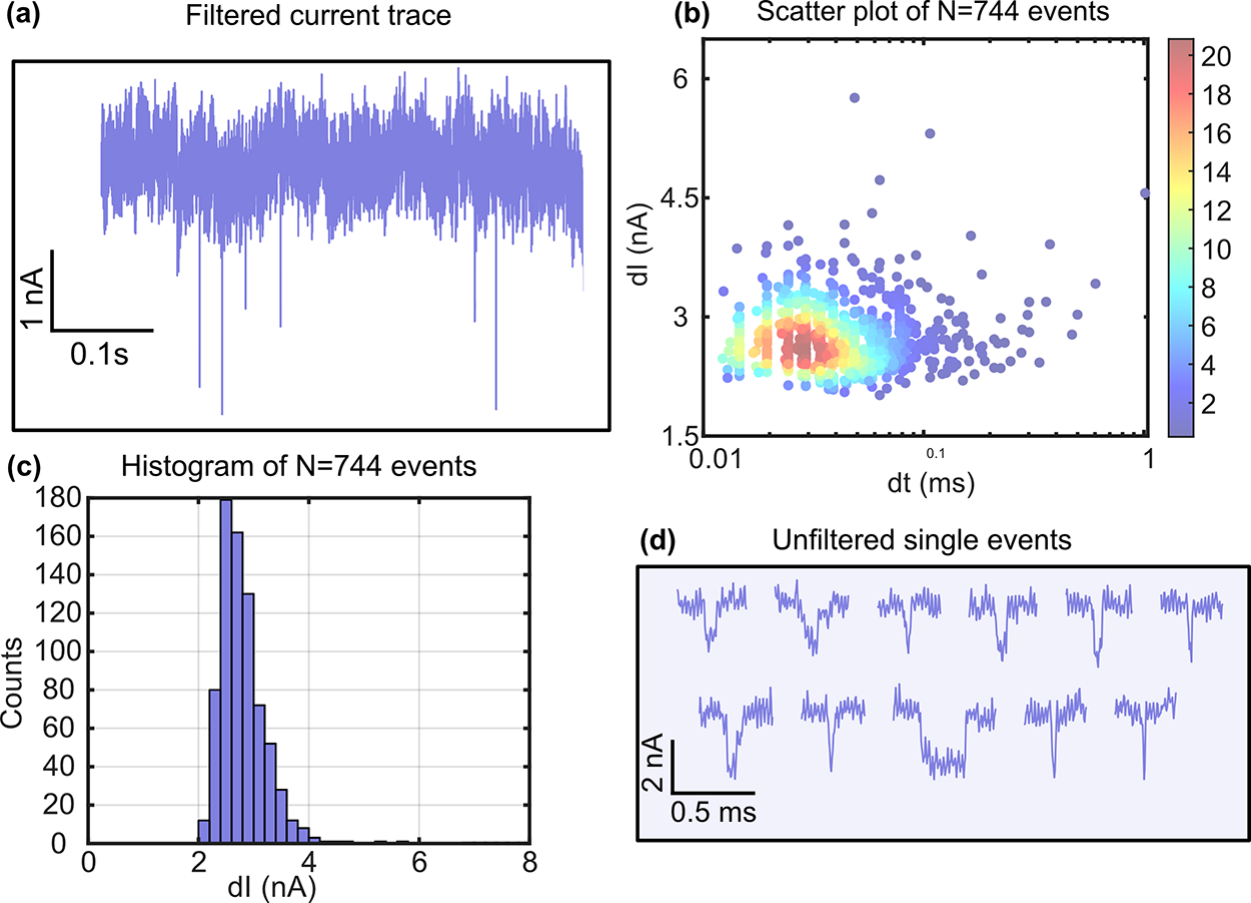
DNA translocation experiments. (a) Current trace for 2 kbp Calf Thymus dsDNA molecules translocating through a ∼ 20 ± 5 nm diameter laser localized CBD nanopore at a bias voltage of 700 mV (200 kHz sampling rate, low pass filtered to 20 kHz for visualization). (b) Heatmap scatter plot of the current blockade (dI) and dwell time (dt) of all 744 events identified at 700 mV. (c) Histogram of current blockade of all events. (d) Selection of extracted individual translocation events.

To show that the nanopores made using our process can function as single-molecule sensors, we performed ionic current sensing of ds-DNA (double-stranded DNA) using 2 kb Calf Thymus DNA (ctDNA) translocating through our pores. Overall, translocation experiments were performed using eight nanopores out of which translocations were observed in three nanopores (labeled Pore 1, Pore 4 and Pore 6). Further details regarding all eight experiments are provided in SI (Table S3, Table S4). A 2s long filtered current trace of 20 nM Calf Thymus DNA translocating through a nanopore (Pore 1) fabricated by CBD after laser exposure at 3.1 mW with an open pore conductance of ∼ 71 nS in 1 M KCl is shown in Figure 6a. The diameter of the nanopore is calculated to be 20 ± 5 nm using the conductance model^54^ assuming that the pore length is equal to the minimum thickness of the membrane’s thinned area. The current blockade amplitude (d*I*) and dwell time of translocation events at a bias voltage, *V*_*bias*_ = 700 *mV*, is shown in Figure 6b. The blockade conductance (Δ*G* = d*I*/*V*_*bias*_) for ds-DNA translocation and the solution conductivity (*s*) can be used to estimate the effective length of the nanopore (*L*) using the well-known conductance blockade model for a cylindrical pore as^54^. Here *d*_*DNA*_ is the diameter of double-stranded DNA and is taken to be 2.2 nm.^31,54^ Using the measured average blockade conductance of 744 events at bias voltage 700 mV extracted from the histogram of current blockade amplitude of all events (Figure 6c), we calculated the effective pore length to be 12 ± 2 nm. A selection of individual translocation events from this nanopore are also shown (Figure 6d). Similar analysis of Calf Thymus DNA translocation using another nanopore (Pore 6, laser exposed at 3.5 mW and open pore conductance after CBD of ∼105 nS; see Figure S8) produces an effective pore length estimate of 11 ± 1 nm. Lambda-DNA translocation experiments were also performed using Pore 1, and the results are presented in SI (Figure S9).

The effective pore lengths of 11–12 nm is significantly less than the 50 nm pristine membrane thickness, as well as the ∼25–34 nm thickness minimum expected for the laser thinned area of the membrane (at 3.1–3.5 mW laser exposure power; Figure 3c) where the nanopore was most likely fabricated. Based on past reports, the relationship between the nanopore length (*L*) and membrane thickness (*t*) is unclear when CBD is used as the fabrication method. This has been best studied in the context of TEM drilled nanopores, where L ∼ t/3 has been reported.^56,57^ Others using CBD have variously reported the L vs t relationship as (1) L ∼ t (for *t* ≤ 10 nm),^21,31,35^ (2) L ∼ t/3 (for *t* ≤ 10 nm and d ≪ t),^55^ and (3) L ∼ t/3 for (*t* > 20 nm and d < t),^29^ also attributing discrepancies from L ∼ t to deviations in the pore geometry from the assumption of a perfect cylinder. Scenario (3) is in good agreement with our finding of *L*∼*t*/(2.9 ± 0.5) for Pore 1 (d ∼ 20 nm) and *L*∼*t*/(2.5 ± 0.2) for Pore 3 (d ∼ 23 nm) for CBD nanopores made on a membrane thinned to ∼ 25–34 nm using a single laser pulse at laser powers of 3.1–3.5 mW. The discrepancy between the membrane thickness inferred from EFTEM data and the pore length calculation from the DNA ruler technique is likely due to a deviation of the real pore shape from the ideal circular cylindrical shape implicitly assumed in the pore conductance model. When a nanopore is fabricated by CBD on a membrane thicker than 25 nm, we see that there is a significant tapering of the pore profile, similar to what is observed in TEM-drilled nanopores.

Since the noise characteristics of a nanopore is important for its function as a sensor, we present the power spectral density (PSD) of five nanopores of nominal diameter 18–33 nm in Figure S7a, b. We observe that the 1/f noise levels vary from sample to sample as expected, and they are spread out over a relatively narrow range. Taking 1 kHz as the bandwidth of importance for 1/f noise, we see that the I_rms_ values of the eight nanopores lie between ∼ 110–380 pA (SI, Table S3). We also compared the noise level in our laser localized nanopores to equivalent nanopores fabricated on an identical substrate but using SiN_*x*_ thinning by reactive ion etching to a thickness of ∼ 27 nm in a 1 μm diameter circular area at the center of the membrane before CBD (Figure S7c). The noise levels were very similar, leading us to conclude that the noise characteristics of our laser localized nanopores were not significantly influenced by the laser exposure process. With the use of undoped silicon wafers,^58^ and dielectric layers between the silicon nitride and silicon,^59^ as well as thinner membranes (<20 nm thick, so that CBD can be performed at lower bias to produce smaller nanopores), and by introducing postfabrication nanopore conditioning approaches like voltage-pulsing, piranha cleaning etc.,^22^ we expect that the overall noise in our laser localized nanopore devices can be improved.

## Conclusions

Using single pulse femtosecond laser exposure of ∼50 nm thick unpatterned silicon nitride membranes, we showed that nanopore fabrication by CBD can be localized to the site of laser exposure. Such localized nanopore formation was possible both above and below the laser power which caused visible ablation of material by laser exposure. Above the ablation threshold, local thinning of the membrane by up to ∼60% of the original thickness over an approximately circular area of ∼150–500 nm diameter, depending on the laser exposure power, was observed. This local thinning was the reason dielectric breakdown occurred preferentially at the site of laser exposure in those samples. Below the ablation threshold, no visible change to the membrane was observable in TEM imaging. Nevertheless, nanopores still formed at the location of laser exposure at these low powers, and we suggest that this was due to internal structural defects (e.g., broken bonds) introduced over a sub-100 nm area of the membrane at the center of the laser beam path. At the lowest evaluated laser power in this study (2.2 mW), which was well below the ablation threshold (∼2.7–2.8 mW), the time for nanopore formation by CBD was still significantly lower than the typical pristine silicon nitride membrane in our study. Therefore, nanopore formation by controlled dielectric breakdown emerges as a capable indirect tool to sensitively probe modification of thin dielectric materials by femtosecond laser exposure, which are challenging to detect even using TEM analysis. Unsurprisingly, at much higher laser powers than the ablation threshold, a single laser pulse punches through the membrane and drills a nanopore, the smallest of which we fabricated had an approximate circular diameter of 90 nm. Regarding membrane thinning above the ablation threshold using single laser pulses, we observed two discontinuous increases in the average thickness of material removed with increasing laser power— first a jump from ∼5% to ∼25% (at 3.1–3.2 mW) of the pristine membrane thickness, and then from ∼30% to ∼100% (3.8–3.9 mW; direct nanopore drilling at the center of the thinned area). Future studies with finer control of laser power at these threshold power values might help determine whether better resolution of material removal along the *z*-axis can be achieved. We also demonstrated ds-DNA translocations using nanopores made using our two-step approach: laser exposure in air followed by nanopore fabrication by CBD. Our two-step technique combines the strengths of direct drilling approaches, where the site of nanopore formation can be precisely determined, and the simplicity of CBD, where nanopores can be formed directly in liquid and whose sizes can be well controlled. We expect our findings to be valuable to further developing our evolving understanding of the nanomachining capabilities of femtosecond pulsed lasers on suspended films, as well as to simplify the manufacturing of more sophisticated solid-state nanopore sensors by enabling localized CBD using a cleanroom-free process carried out in ambient conditions.

## Methods

### Fabrication of Silicon Nitride Membranes

A 100 mm diameter, 380 ± 15 um thick double-side polished single-crystalline silicon wafer (100) with boron doping and a resistivity of 1–10 Ω-cm, with 50 ± 5 nm of low-pressure chemical vapor deposition (LPCVD) Si-rich silicon nitride (SiN_*x*_) was used as a starting substrate for microfabrication. Using photolithography, silicon nitride etching by reactive ion etching (RIE), and wet etching through the Si wafer by 30% potassium hydroxide (KOH) at 80 °C with the silicon nitride as a hard mask, suspended square-shaped silicon nitride (SiNx) membranes with dimensions 40 μm × 40 μm were fabricated. As a last step, the membranes were cleaned with a 3 H_2_SO_4_: 1 H_2_O_2_ piranha solution for 10 min to remove organic residues.

### Femtosecond Laser Exposures

Single pulse laser exposure of silicon nitride membranes was carried out with a subpicosecond diode-pumped solid-state laser with a fundamental wavelength of 1040 nm and second harmonic wavelength of 520 nm (Spirit 1040–4-SHG, Spectra-Physics of Newport Corporation). The laser exposures were performed using a wavelength of 520 nm, pulse duration of 293 fs, and repetition rate of 1 MHz. An absorptive neutral density (NE10A, Thorlabs) filter with an Optical Density (OD) of 1.0 was placed in the optical path to reduce the optical power of the incident laser beam by a factor of 10. Set powers of 170–230 mW were used to achieve 2.2–3.8 mW average powers as measured after the objective. A mechanical shutter with millisecond accuracy and a pulse-picker divider controlled the number of pulses that irradiated the membrane– which was one pulse for all samples in this study. An absorptive neutral density (ND) filter with an optical density (OD) of 1.0 was used to reduce the optical power of the incident laser beam. The objective (Olympus Plan Achromat RMS 40X) with a numerical aperture of 0.65 was used to focus the beam on the membrane. Before the silicon nitride exposures, we measured the average laser power output after the objective with a silicon optical power detector (918D-SL-OD3R, Newport). It is the laser power after the objective which are relevant to interpret our results, and therefore it is these powers which are mentioned in the results presented throughout this study. We investigated a range of laser powers between 2 and 4 mW. To convert the average laser power values to the laser pulse energy values, we can divide each power value by the repetition rate we used for the experiments. Hence, the corresponding laser pulse energy values are 2 to 4 nJ. The chip was placed on a 3-axis linear motorized stage (XMS100, Newport), and the beam was focused on the substrate. To avoid any unwanted damage of the suspended membrane area, we performed the laser focusing procedure on the supported silicon nitride surface beside the membrane. Subsequently, during the nanopore fabrication with CBD or pulsed CBD, the PDMS adaptors cover all sites on which the laser focusing procedure was performed. To ensure a correct laser focus on the membrane during the membrane exposure, we used the three-point substrate leveling routine built into the software of our motorized stage. In this method three in-focus points around the membrane area are selected, each with a distance of about 700 μm from the membrane edge. Next, the x, y and z coordinates of the three focus points are used to perform substrate leveling, assuming a planar surface. The entire focusing procedure takes approximately 10 min per sample in our system.

### Nanopore Fabrication by Controlled Breakdown

All samples consisted of chips that were ∼ 5 × 5 mm^2^ in size, with a single suspended membrane (40 μm × 40 μm square) at the center. Chips were cleaned in a UVO cleaner (Jelight Model 30) for 5 min to increase hydrophilicity just before nanopore fabrication. The chip was first sealed between two PDMS adaptors, and this sandwich was placed inside a custom-designed two-part 3D-printed flow cell printed using a Form 3+ printer (Formlabs, USA; Clear Resin v4). The entire assembly was held together using metal screws.^22^ CBD was performed in a buffered solution of 1 M KCl and 10 mM HEPES at pH 8.0 and ionic conductivity of 11.0 S/m by filling reservoirs on both sides of the flow cell with the buffer. Ag/AgCl electrodes were used to make terminal electrical contacts to the chip inside a Faraday cage.

Nanopore fabrication was performed using two different setups to implement the two kinds of dielectric breakdown processes, (1) controlled breakdown (CBD) and (2) pulsed CBD, used in this study. These setups follow standard procedures reported by various groups previously,^21,22,29,55^ and details of our implementation are described in brief here. During (1) CBD, a fixed voltage bias is applied across a membrane until an abrupt current increase is observed, indicating the formation of a nanopore. At this point, the bias is immediately switched off. During (2) pulsed CBD, two pulsed voltage levels are used— a high voltage (V_Hi_) for breakdown and a low voltage (V_Lo_) to sense— and was developed as a refinement of the CBD process to fabricate sub-10 nm nanopores.

We performed (1) CBD using a DC Voltage Source (Keithley 2220–30–1) to apply the voltage bias (up to 30 V) and measured current using a trans-impedance amplifier (DLPCA 200; Femto GmbH) connected to a DC SMU (Ossila X200). All instruments were controlled using custom LabView scripts.

We performed (2) pulsed CBD using a DC SMU (Keithley 2450), which was used to both apply the pulsed voltage bias and measure current. The nanopore fabrication process was controlled using a combination of scripts running on the onboard logic processor of the DC SMU and those running on Matlab to collect and process the acquired data. The parameters we used in the pulsed CBD process were V_Hi_ = 20–27.5 V, T_Hi_ = 300 ms, V_Lo_ = 300 mV, and T_Lo_ = 3 s. Here, T_Hi_ and T_Lo_ are the time durations for the high- and low-voltage pulses, respectively.

### Optical Visualization of Nanopores

The location of nanopore formation on a membrane was visualized using fluorescent microscopy performed using a confocal inverted microscope (Zeiss LSM900 with airy2 detector) and a 40x water immersion objective lens (Zeiss C-Apochromat 40*x*/1.2W). The nanopore chip was glued (Loctite 420) to a custom-designed 3D-printed flow cell (Form 3+; Clear Resin v4), and the assembly was sealed with a cover glass (24 mm × 50 mm, #0) for imaging (Figure 2c), with the membrane side of the chip (the other side being the backside of the chip with the Si cavity) facing the objective. For the nanopore visualization experiments, the chamber on the membrane side was filled with 100 mM CaCl_2,_ 1 M KCl, 40 mM Tris-HCl, 1 mM EDTA solution, and the chamber on the backside with 500 nM Fluo-4 (F14200, ThermoFisher; Pentapotassium salt, cell impermeant) in 1 M KCl, 40 mM Tris-HCl, 1 mM EDTA buffer. These are the same buffers used for a similar application by Zrehen et al.^29^ Ag/AgCl wire electrodes were used to make electrical connections to the two chambers, and a DC SMU (Ossila X200) was used to apply the voltage bias during imaging. The electrode on the backside chamber was biased, and the membrane side was held at the ground in all the visualization experiments. Image processing and analysis of the visualization experiments were performed using FIJI.

### TEM Imaging

TEM investigations were performed in a Zeiss LIBRA 200 FEG operated at 200 kV. Bright Field TEM images were recorded at various magnifications on a GATAN slow scan CCD camera. The Zeiss LIBRA is equipped with a corrected in-column Omega filter, which was employed to acquire energy-filtered TEM (EFTEM) images and electron energy loss spectra (EELS). Thickness maps were acquired using the *t*/λ technique, which allows the generation of a map of the relative thickness (*t*) normalized by the mean free path for inelastic scattering (λ). EELS spectra were recorded by projecting the energy dispersive plane of the Omega filter on the CCD camera at suitable postmagnification.

### Translocation of dsDNA

Double-stranded DNA (UltraPure Calf Thymus DNA Solution, ThermoFisher) with a final concentration of 20 nM in a buffered solution of 1 M KCl and 10 mM HEPES at pH 8.0 and ionic conductivity of 11.3 S/m was added to the cis chamber (membrane side of the chip). The trans chamber of the flow cell was positively biased, driving the DNA through the nanopore from cis to trans. The translocation data was recorded at an applied bias of 500 mV and 700 mV at a sampling rate of 200 kHz using eNPR (Nanopore Reader, Elements SRL, Italy). The data as acquired (unfiltered) was analyzed using EventPro 3.0 to determine the conductance blockade amplitudes and dwell times for the translocation events.^60^

## Supporting Information Available

Fluorescent optical visualization of the three nanopores that failed to be localized, fluorescent optical visualization images of selected membranes, (Supplementary Note 1) formation of multiple nanopores after CBD on two silicon nitride membranes laser-exposed in a 3 × 3 array pattern, BF-TEM images of the membranes around the ablation threshold- 2.7 and 2.8 mW, EFTEM maps at various laser powers, *I*–*V* sweeps of nanopores fabricated by pulsed CBD, summary of nanopores size change rate per day for 10 samples, eight laser localized CBD nanopores with which DNA translocation experiments were performed, summary of DNA translocation data obtained from three nanopores, power spectrum density (PSD) of the ionic current obtained from various nanopores in this study, DNA translocation experiments using Pore 6, and lambda DNA translocation experiments (PDF)

## References

[ref1] Xue L.; Yamazaki H.; Ren R.; Wanunu M.; Ivanov A. P.; Edel J. B. Solid-State Nanopore Sensors. Nature Reviews Materials 2020, 5, 931–951. 10.1038/s41578-020-0229-6.

[ref2] Wanunu M.; Sutin J.; Meller A. DNA Profiling Using Solid-State Nanopores: Detection of DNA-Binding Molecules. Nano Lett. 2009, 9, 3498–3502. 10.1021/nl901691v. 19585985 PMC2871189

[ref3] Yusko E. C.; Johnson J. M.; Majd S.; Prangkio P.; Rollings R. C.; Li J.; Yang J.; Mayer M. Controlling Protein Translocation Through nanopores With Bio-inspired Fluid Walls. Nat. Nanotechnol. 2011, 6, 253–260. 10.1038/nnano.2011.12. 21336266 PMC3071889

[ref4] Hu R.; Rodrigues J. V.; Waduge P.; Yamazaki H.; Cressiot B.; Chishti Y.; Makowski L.; Yu D.; Shakhnovich E.; Zhao Q.; Wanunu M. Differential Enzyme Flexibility Probed Using Solid-State Nanopores. ACS Nano 2018, 12, 4494–4502. 10.1021/acsnano.8b00734. 29630824 PMC9016714

[ref5] Yang W.; Restrepo-Pérez L.; Bengtson M.; Heerema S. J.; Birnie A.; Van Der Torre J.; Dekker C. Detection of CRISPR-dCas9 on DNA with Solid-State Nanopores. Nano Lett. 2018, 18, 6469–6474. 10.1021/acs.nanolett.8b02968. 30187755 PMC6187524

[ref6] Sandler S. E.; Weckman N. E.; Yorke S.; Das A.; Chen K.; Gutierrez R.; Keyser U. F. Sensing the DNA-Mismatch Tolerance of Catalytically Inactive Cas9 via Barcoded DNA Nanostructures in Solid-State Nanopores. Nat. Biomed. Eng. 2023, 8, 325 10.1038/s41551-023-01078-2. 37550424 PMC10963265

[ref7] Chen K.; Choudhary A.; Sandler S. E.; Maffeo C.; Ducati C.; Aksimentiev A.; Keyser U. F. Super-Resolution Detection of DNA Nanostructures Using a Nanopore. Adv. Mater. 2023, 35, 2207434 10.1002/adma.202207434. 36630969

[ref8] Chen K.; Jou I.; Ermann N.; Muthukumar M.; Keyser U. F.; Bell N. A. W. Dynamics of Driven Polymer Transport Through a Nanopore. Nat. Phys. 2021, 17, 1043–1049. 10.1038/s41567-021-01268-2.

[ref9] Schmid S.; Stömmer P.; Dietz H.; Dekker C. Nanopore Electro-Osmotic Trap for the Label-Free Study of Single Proteins and Their Conformations. Nat. Nanotechnol. 2021, 16, 1244–1250. 10.1038/s41565-021-00958-5. 34462599

[ref10] Yokota K.; Tsutsui M.; Taniguchi M. Electrode-Embedded Nanopores for Label-Free Single-Molecule Sequencing by Electric Currents. RSC Adv. 2014, 4, 15886–15899. 10.1039/C4RA00933A.

[ref11] Pud S.; Verschueren D.; Vukovic N.; Plesa C.; Jonsson M. P.; Dekker C. Self-Aligned Plasmonic Nanopores by Optically Controlled Dielectric Breakdown. Nano Lett. 2015, 15, 7112–7117. 10.1021/acs.nanolett.5b03239. 26333767 PMC4859154

[ref12] Assad O. N.; Gilboa T.; Spitzberg J.; Juhasz M.; Weinhold E.; Meller A. Light-Enhancing Plasmonic-Nanopore Biosensor for Superior Single-Molecule Detection. Adv. Mater. 2017, 29, 1605442 10.1002/adma.201605442. 28026129

[ref13] Larkin J.; Henley R. Y.; Jadhav V.; Korlach J.; Wanunu M. Length-Independent DNA Packing into Nanopore Zero-Mode Waveguides for Low-Input DNA Sequencing. Nat. Nanotechnol. 2017, 12, 1169–1175. 10.1038/nnano.2017.176. 28892102 PMC5718969

[ref14] Storm A. J.; Chen J. H.; Ling X. S.; Zandbergen H. W.; Dekker C. Fabrication of Solid-State Nanopores with Single-Nanometre Precision. Nat. Mater. 2003, 2, 537–540. 10.1038/nmat941. 12858166

[ref15] Li J.; Stein D.; Mcmullan C.; Branton D.; Aziz M. J.; Golovchenko J. A. Ion-Beam Sculpting at Nanometre Length Scales. Nature 2001, 412, 166–169. 10.1038/35084037. 11449268

[ref16] Chou Y. C.; Masih Das P.; Monos D. S.; Drndic M. Lifetime and Stability of Silicon Nitride Nanopores and Nanopore Arrays for Ionic Measurements. ACS Nano 2020, 14, 6715–6728. 10.1021/acsnano.9b09964. 32275381 PMC9547353

[ref17] Vlassiouk I.; Apel P. Y.; Dmitriev S. N.; Healy K.; Siwy Z. S. Versatile ultrathin nanoporous silicon nitride membranes. Proc. Natl. Acad. Sci. U. S. A. 2009, 106 (50), 21039–21044. 10.1073/pnas.0911450106. 19948951 PMC2795523

[ref18] Verschueren D. V.; Yang W.; Dekker C. Lithography-Based Fabrication of Nanopore Arrays in Freestanding SiN and Graphene Membranes. Nanotechnology 2018, 29, 145302 10.1088/1361-6528/aaabce. 29384130 PMC5997186

[ref19] Yamazaki H.; Hu R.; Zhao Q.; Wanunu M. Photothermally Assisted Thinning of Silicon Nitride Membranes for Ultrathin Asymmetric Nanopores. ACS Nano 2018, 12, 12472–12481. 10.1021/acsnano.8b06805. 30457833

[ref20] Zvuloni E.; Zrehen A.; Gilboa T.; Meller A. Fast and Deterministic Fabrication of Sub-5 Nanometer Solid-State Pores by Feedback-Controlled Laser Processing. ACS Nano 2021, 15, 12189–12200. 10.1021/acsnano.1c03773. 34219449 PMC8320231

[ref21] Kwok H.; Briggs K.; Tabard-Cossa V. Nanopore Fabrication by Controlled Dielectric Breakdown. PLoS One 2014, 9, e92880 10.1371/journal.pone.0092880. 24658537 PMC3962464

[ref22] Waugh M.; Briggs K.; Gunn D.; Gibeault M.; King S.; Ingram Q.; Jimenez A. M.; Berryman S.; Lomovtsev D.; Andrzejewski L.; et al. Solid-State Nanopore Fabrication by Automated Controlled Breakdown. Nat. Protoc 2020, 15, 122–143. 10.1038/s41596-019-0255-2. 31836867

[ref23] Kennedy E.; Dong Z.; Tennant C.; Timp G. Reading the Primary Structure of a Protein with 0.07 nm3 Resolution Using a Subnanometre-Diameter Pore. Nat. Nanotechnol. 2016, 11, 968–976. 10.1038/nnano.2016.120. 27454878

[ref24] Gilboa T.; Zvuloni E.; Zrehen A.; Squires A. H.; Meller A. Automated, Ultra-Fast Laser-Drilling of Nanometer Scale Pores and Nanopore Arrays in Aqueous Solutions. Adv. Funct. Mater. 2020, 30, 1900642 10.1002/adfm.201900642.

[ref25] Yanagi I.; Akahori R.; Takeda K.-I. Stable Fabrication of a Large Nanopore by Controlled Dielectric Breakdown in a High-pH Solution for the Detection of Various-Sized Molecules. Sci. Rep. 2019, 9, 13143 10.1038/s41598-019-49622-y. 31511597 PMC6739384

[ref26] Nicoli F.; Verschueren D.; Klein M.; Dekker C.; Jonsson M. P. DNA Translocations through Solid-State Plasmonic Nanopores. Nano Lett. 2014, 14, 6917–6925. 10.1021/nl503034j. 25347403 PMC4264857

[ref27] Krishnakumar P.; Gyarfas B.; Song W.; Sen S.; Zhang P.; Krstić P.; Lindsay S. Slowing DNA Translocation through a Nanopore Using a Functionalized Electrode. ACS Nano 2013, 7, 10319–10326. 10.1021/nn404743f. 24161197 PMC3875158

[ref28] Ivanov A. P.; Freedman K. J.; Kim M. J.; Albrecht T.; Edel J. B. High Precision Fabrication and Positioning of Nanoelectrodes In a Nanopore. ACS Nano 2014, 8, 1940–1948. 10.1021/nn406586m. 24446951

[ref29] Zrehen A.; Gilboa T.; Meller A. Real-time Visualization and Sub-Diffraction Limit Localization of Nanometer-Scale Pore Formation by Dielectric Breakdown. Nanoscale 2017, 9, 16437–16445. 10.1039/C7NR02629C. 29058736

[ref30] Zhang Y.; Ma D.; Gu Z.; Zhan L.; Sha J. Fast Fabrication of Solid-State Nanopores for DNA Molecule Analysis. Nanomaterials 2021, 11, 2450 10.3390/nano11092450. 34578767 PMC8468320

[ref31] Carlsen A. T.; Briggs K.; Hall A. R.; Tabard-Cossa V. Solid-State Nanopore Localization by Controlled Breakdown of Selectively Thinned Membranes. Nanotechnology 2017, 28, 085304–085304. 10.1088/1361-6528/aa564d. 28045003 PMC5408306

[ref32] Ying C.; Houghtaling J.; Eggenberger O. M.; Guha A.; Nirmalraj P.; Awasthi S.; Tian J.; Mayer M. Formation of Single Nanopores with Diameters of 20–50 nm in Silicon Nitride Membranes Using Laser-Assisted Controlled Breakdown. ACS Nano 2018, 12, 11458–11470. 10.1021/acsnano.8b06489. 30335956

[ref33] Zhang Y.; Miyahara Y.; Derriche N.; Yang W.; Yazda K.; Capaldi X.; Liu Z.; Grutter P.; Reisner W. Nanopore Formation via Tip-Controlled Local Breakdown Using an Atomic Force Microscope. Small Methods 2019, 3 (7), 1900147 10.1002/smtd.201900147.

[ref34] Fried J. P.; Swett J. L.; Nadappuram B. P.; Fedosyuk A.; Gee A.; Dyck O. E.; Yates J. R.; Ivanov A. P.; Edel J. B.; Mol J. A. Localised solid-state nanopore fabrication via controlled breakdown using on-chip electrodes. Nano Res. 2022, 15 (11), 9881–9889. 10.1007/s12274-022-4535-8.

[ref35] Briggs K.; Kwok H.; Tabard-Cossa V. Automated Fabrication of 2-nm Solid-State Nanopores for Nucleic Acid Analysis. Small 2014, 10, 2077–2086. 10.1002/smll.201303602. 24585682

[ref36] Tang Z.; Dong M.; He X.; Guan W. On Stochastic Reduction in Laser-Assisted Dielectric Breakdown for Programmable Nanopore Fabrication. ACS Appl. Mater. Interfaces 2021, 13, 13383–13391. 10.1021/acsami.0c23106. 33705089

[ref37] Bandara Y. M. N. D. Y.; Karawdeniya B. I.; Dutt S.; Kluth P.; Tricoli A. Nanopore Fabrication Made Easy: A Portable, Affordable Microcontroller-Assisted Approach for Tailored Pore Formation via Controlled Breakdown. Anal. Chem. 2024, 96, 2124–2134. 10.1021/acs.analchem.3c04860. 38277343

[ref38] Liao C.; Wuethrich A.; Trau M. A Material Odyssey for 3D Nano/Microstructures: Two Photon Polymerization Based Nanolithography in Bioapplications. Appl. Mater. Today 2020, 19, 100635 10.1016/j.apmt.2020.100635.

[ref39] Chen Q.; Liu Z. Fabrication and Applications of Solid-State Nanopores. Sensors 2019, 19, 1886 10.3390/s19081886. 31010038 PMC6515193

[ref40] Zhang B.; Wang Z.; Tan D.; Qiu J. Ultrafast Laser-Induced Self-Organized Nanostructuring in Transparent Dielectrics: Fundamentals and Applications. PhotoniX 2023, 4, 24 10.1186/s43074-023-00101-8.

[ref41] Akkanen S. T. M.; Fernandez H. A.; Sun Z. Optical Modification of 2D Materials: Methods and Applications. Adv. Mater. 2022, 34, 2110152 10.1002/adma.202110152. 35139583

[ref42] Enrico A.; Hartwig O.; Dominik N.; Quellmalz A.; Gylfason K. B.; Duesberg G. S.; Niklaus F.; Stemme G. Ultrafastand Resist-Free Nanopatterning of 2D Materials by Femtosecond Laser Irradiation. ACS Nano 2023, 17 (9), 8041–8052. 10.1021/acsnano.2c09501. 37074334 PMC10173691

[ref43] Lin Z.; Liu K.; Cao T.; Hong M. Microsphere Femtosecond Laser Sub-50 nm Structuring in Far Field via Non-Linear Absorption. Opto-Electron. Adv. 2023, 6, 230029 10.29026/oea.2023.230029.

[ref44] Gil-Villalba A.; Meyer R.; Giust R.; Rapp L.; Billet C.; Courvoisier F. Single Shot Femtosecond Laser Nano-Ablation of CVD Monolayer Graphene. Sci. Rep. 2018, 8, 14601 10.1038/s41598-018-32957-3. 30279433 PMC6168448

[ref45] Mentel K. K.; Manninen J.; Hiltunen V.-M.; Myllyperkiö P.; Johansson A.; Pettersson M. Shaping graphene with optical forging: from a single blister to complex 3D structures. Nanoscale Advances 2021, 3, 1431–1442. 10.1039/D0NA00832J. 36132861 PMC9419103

[ref46] Johansson A.; Myllyperkiö P.; Koskinen P.; Aumanen J.; Koivistoinen J.; Tsai H.-C.; Chen C.-H.; Chang L.-Y.; Hiltunen V.-M.; Manninen J. J.; et al. Optical Forging of Graphene into Three-Dimensional Shapes. Nano Lett. 2017, 17 (10), 6469–6474. 10.1021/acs.nanolett.7b03530. 28926715

[ref47] Uesugi Y.; Kozawa Y.; Sato S. Nanoprocessing of Free-Standing Thin Films by Ultrafast Laser Ablation. In Laser-based Micro- and Nanoprocessing XV; SPIE: 2021.

[ref48] Uesugi Y.; Fukushima R.; Kozawa Y.; Sato S. Ultrafast Laser Ablation of 10-nm Self-Supporting membranes by two-beam interference processing. Opt. Express 2020, 28, 26200 10.1364/OE.400941. 32906896

[ref49] Morimoto Y.; Roland I.; Rennesson S.; Semond F.; Boucaud P.; Baum P. Laser Damage of Free-Standing Nanometer Membranes. J. Appl. Phys. 2017, 122, 215303 10.1063/1.5004081.

[ref50] Xie X.; Nikbakht R.; Couillard M.; St-Gelais R.; Weck A. Laser Machining of Free-Standing Silicon Nitride Membranes. J. Mater. Process. Technol. 2023, 318, 118001 10.1016/j.jmatprotec.2023.118001.

[ref51] Uesugi Y.; Miwa T.; Kadoguchi N.; Kozawa Y.; Sato S. Multi-Beam Ultrafast Laser Processing of Free-Standing Nanofilms. Appl. Phys. A: Mater. Sci. Process. 2023, 129, 101 10.1007/s00339-022-06361-8.

[ref52] Briggs K.; Charron M.; Kwok H.; Le T.; Chahal S.; Bustamante J.; Waugh M.; Tabard-Cossa V. Kinetics of Nanopore Fabrication During Controlled Breakdown of Dielectric Membranes in Solution. Nanotechnology 2015, 26, 084004 10.1088/0957-4484/26/8/084004. 25648336

[ref53] Wang Y.; Ying C.; Zhou W.; De Vreede L.; Liu Z.; Tian J. Fabrication of multiple nanopores in a SiNx membrane via controlled breakdown. Sci. Rep. 2018, 8 (1), 1234 10.1038/s41598-018-19450-7. 29352158 PMC5775244

[ref54] Kowalczyk S. W.; Grosberg A. Y.; Rabin Y.; Dekker C. Modeling the Conductance and DNA Blockade of Solid-State Nanopores. Nanotechnology 2011, 22, 315101 10.1088/0957-4484/22/31/315101. 21730759

[ref55] Yanagi I.; Akahori R.; Hatano T.; Takeda K.-I. Fabricating Nanopores with Diameters of Sub-1 to 3 nm Using Multilevel Pulse-Voltage Injection. Sci. Rep. 2015, 4, 5000 10.1038/srep05000. PMC4028839 24847795

[ref56] Wanunu M.; Dadosh T.; Ray V.; Jin J.; Mcreynolds L.; Drndić M. Rapid electronic detection of probe-specific microRNAs using thin nanopore sensors. Nat. Nanotechnol. 2010, 5 (11), 807–814. 10.1038/nnano.2010.202. 20972437

[ref57] Venta K.; Shemer G.; Puster M.; Rodríguez-Manzo J. A.; Balan A.; Rosenstein J. K.; Shepard K.; Drndić M. Differentiation of Short, Single-Stranded DNA Homopolymers in Solid-State Nanopores. ACS Nano 2013, 7 (5), 4629–4636. 10.1021/nn4014388. 23621759 PMC3724363

[ref58] Waggoner P. S.; Kuan A. T.; Polonsky S.; Peng H.; Rossnagel S. M. Increasing the speed of solid-state nanopores. J. Vac. Sci. Technol., B 2011, 29 (3), 032206 10.1116/1.3585536.

[ref59] Balan A.; Machielse B.; Niedzwiecki D.; Lin J.; Ong P.; Engelke R.; Shepard K. L.; Drndić M. Improving Signal-to-Noise Performance for DNA Translocation in Solid-State Nanopores at MHz Bandwidths. Nano Lett. 2014, 14 (12), 7215–7220. 10.1021/nl504345y. 25418589

[ref60] Bandara Y. M. N. D. Y.; Saharia J.; Karawdeniya B. I.; Kluth P.; Kim M. J. Nanopore Data Analysis: Baseline Construction and Abrupt Change-Based Multilevel Fitting. Anal. Chem. 2021, 93, 11710–11718. 10.1021/acs.analchem.1c01646. 34463103

