# Supplementary material for: Localized Nanopore Fabrication in Silicon Nitride Membranes by Femtosecond Laser Exposure and Subsequent Controlled Breakdown
Source: ACS Appl Mater Interfaces. 2025 Jan 27;17(5):8737–48. doi: 10.1021/acsami.5c00255 (PMC11803561; doi:10.1021/acsami.5c00255)
Supplement: Supplementary file 1 — am5c00255_si_001.pdf [file am5c00255_si_001.pdf]

Supplementary Information for:

Localized nanopore fabrication in silicon nitride membranes by femtosecond laser exposure and subsequent controlled breakdown

Chrysovalantou V. Leva[1], *Saumey Jain*[1,2], *Kevin Kistermann*[3], *Kasumi Sakurai*[1], *Göran Stemme*[1], *Anna Herland*[2,4], *Joachim Mayer*[3], *Frank Niklaus*[+,1] *and Shyamprasad N. Raja*[*,1]

[1] Division of Micro and Nanosystems (MST), School of Electrical Engineering and Computer Science (EECS), KTH Royal Institute of Technology, Stockholm SE-10044, Sweden

[2] Division of Nanobiotechnology, SciLifeLab, Department of Protein Science, School of Engineering Sciences in Chemistry, Biotechnology and Health (CBH), KTH Royal Institute of Technology, Stockholm SE-10044, Sweden

[3] Central Facility for Electron Microscopy (GFE), RWTH Aachen University, Aachen 52056, Germany

[4] AIMES, Center for Integrated Medical and Engineering Science, Department of Neuroscience, Karolinska Institute, Solna 17177, Sweden

Email: [*]Shyamprasad N. Raja – shnr@kth.se, [+]Frank Niklaus – frank@kth.se

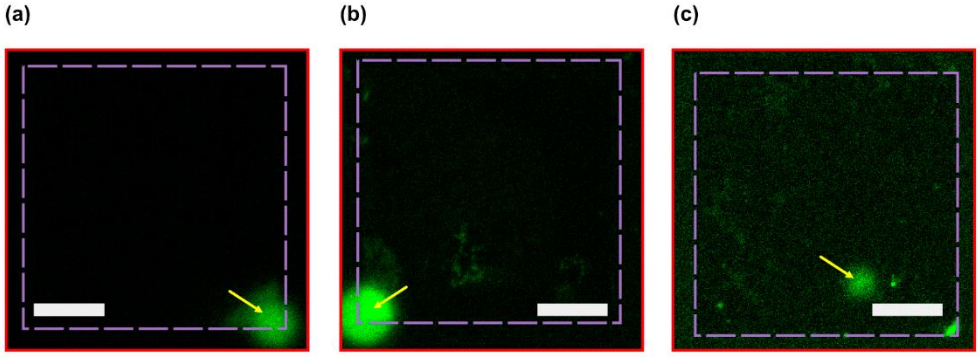

Figure S1. Fluorescent optical visualization of the three nanopores which failed to be localized to the spot of laser exposure. (a), (b) Nanopore formed at the edges of the membranes, and in (c) the nanopore formed near the edge. Purple box shows the outline of the membrane. Laser exposure was performed at the center of the membrane in all three cases. The time to breakdown was between 15–21 seconds, significantly shorter than the breakdown time of most of the CBD membranes presented in Fig 2b. The laser powers used were (a) 2.3 mW, (b) 2.8 mW, and (c) 3.2 mW. We speculate that nanopores formed at these positions instead of the site of laser exposure because of more prominent pre-existing defects on the membrane at the site of nanopore formation as compared to those defects caused by laser exposure, probably introduced during the membrane fabrication process. Scale bar is 10 μm.

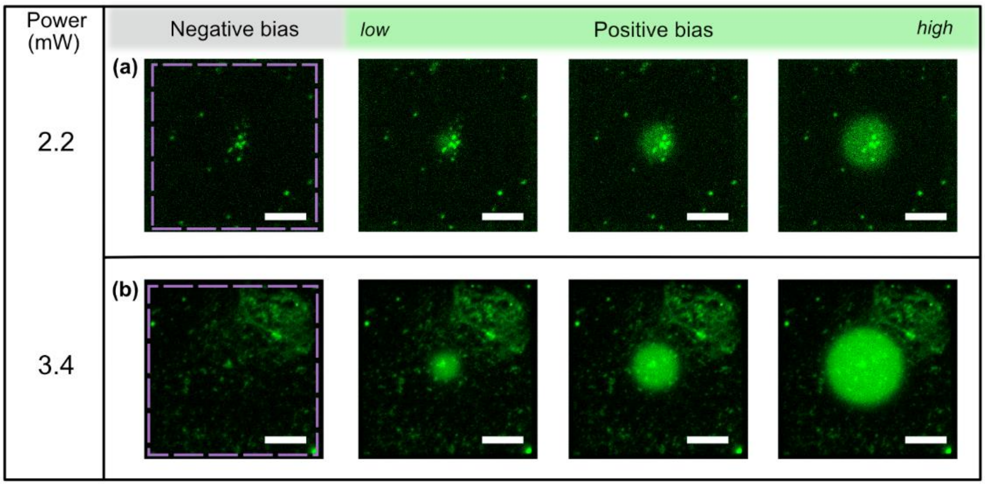

Figure S2. Fluorescent optical visualization images of selected membranes for negative and positive polarity bias voltage application. (a) The membrane is exposed to a laser power of 2.2 mW in the center of the membrane. The bright spots that are observed on the surface of the membrane, and most of them accumulated around the center of the membrane, remain the same as we increased the voltage bias from 0 to positive bias. On the other hand, in the nanopore location, the fluorescent emission is responding and increasing while the voltage bias is increasing. (b) The membrane is exposed to a laser power of 3.4 mW in the center of the membrane. Here, we can observe bright residues/areas around the membrane and next to the upper right corner of the membrane. Similar to (a), except for the area around the membrane where the fluorescent signal is increasing while the voltage bias is increasing, the rest of the bright spots/areas remain the same. The scale bar is 10 μm.

Supplementary Note 1. Formation of multiple nanopores after CBD on two silicon nitride membranes laser-exposed in a 3x3 array pattern. By laser exposing a 3x3 array of spots at a 5 µm pitch using identical laser conditions (2.7 mW at membrane 1 and 3.1 mW at membrane 2), we fabricated multiple nanopores simultaneously on a single membrane using CBD. Two instances are shown in Fig. S3, where two and four nanopores were formed on a single membrane, respectively. Multiple nanopores forming simultaneously at similar breakdown times at well-separated locations on the same membrane indicate the similarity of the damage sites created using nominally identical laser exposure conditions. On the other hand, the fact that not all nine laser-exposed spots of the array produce a nanopore also shows the variability of the generated damage and illustrates the stochastic nature of nanopore formation by the CBD process. When a membrane is locally thinned in multiple locations before the nanopore formation, the voltage termination delay after the first pore formation can result in the formation of more than one pore on the thinned areas[1]. In our case, membranes 1 and 2 have been exposed to a 3x3 array, which translates to 9 possible positions that can facilitate nanopore formation. In contrast with the single nanopores formation where the voltage application is terminated in less than 10 ms after the nanopore formation, for membranes 1 and 2 (patterned with 3x3 arrays), the delay between the nanopore formation moment and the voltage termination is more than 1 second. For Membrane 1, voltage termination occurred 3.6 seconds after the first nanopore formation, whereas for Membrane 2, the voltage termination was 11.4 seconds after the first pore formation. In membrane 2, the combination of higher laser power at the laser exposure (3.1 mW) leads to thinner silicon nitride areas, and the longer voltage termination delay results in the fabrication of nanopores in 4 out of 9 thinned positions. Membrane 1 is exposed to 2.7 mW laser power, and the termination delay after the first pore formation was shorter compared to membrane 2, so we got nanopores at 2 out of 9 thinned positions. It is likely that more pores would have formed at the other laser-exposed spots of the arrays had we allowed the CBD process to continue even longer; however, in this study, we did not investigate this aspect.

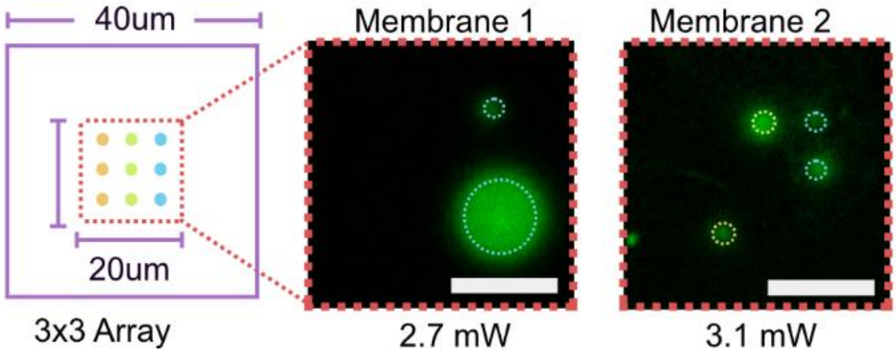

Figure S3. Fluorescent images show the formation of multiple nanopores after CBD on two membranes laser-exposed in a 3x3 array pattern. CBD was stopped soon after the first pore formation was detected, but multiple nanopores were still produced. Laser exposure sites from the three columns are represented by three different colors: orange(left), green(middle) and blue(right). The scale bar is 10 µm.

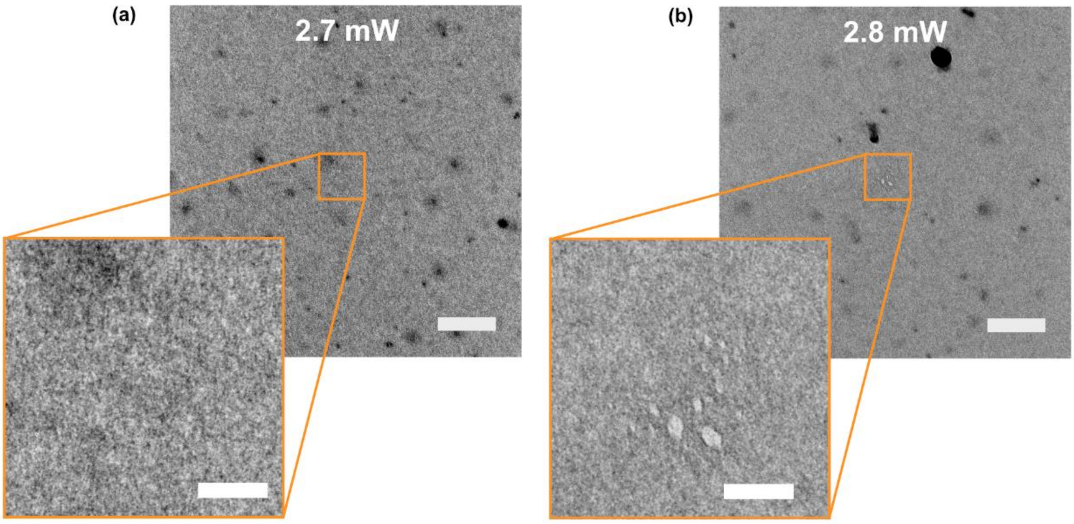

Figure S4. BF-TEM images of the membrane surface around the ablation threshold. (a) At 2.7 mW there was no observable modification of the membrane. (b) At 2.8mW we observed a disconnected nanoscopic cluster of laser thinned areas at the site of the laser exposure. The thinning is seen as a brighter contrast in BF-TEM. Scale bar is 200 nm.

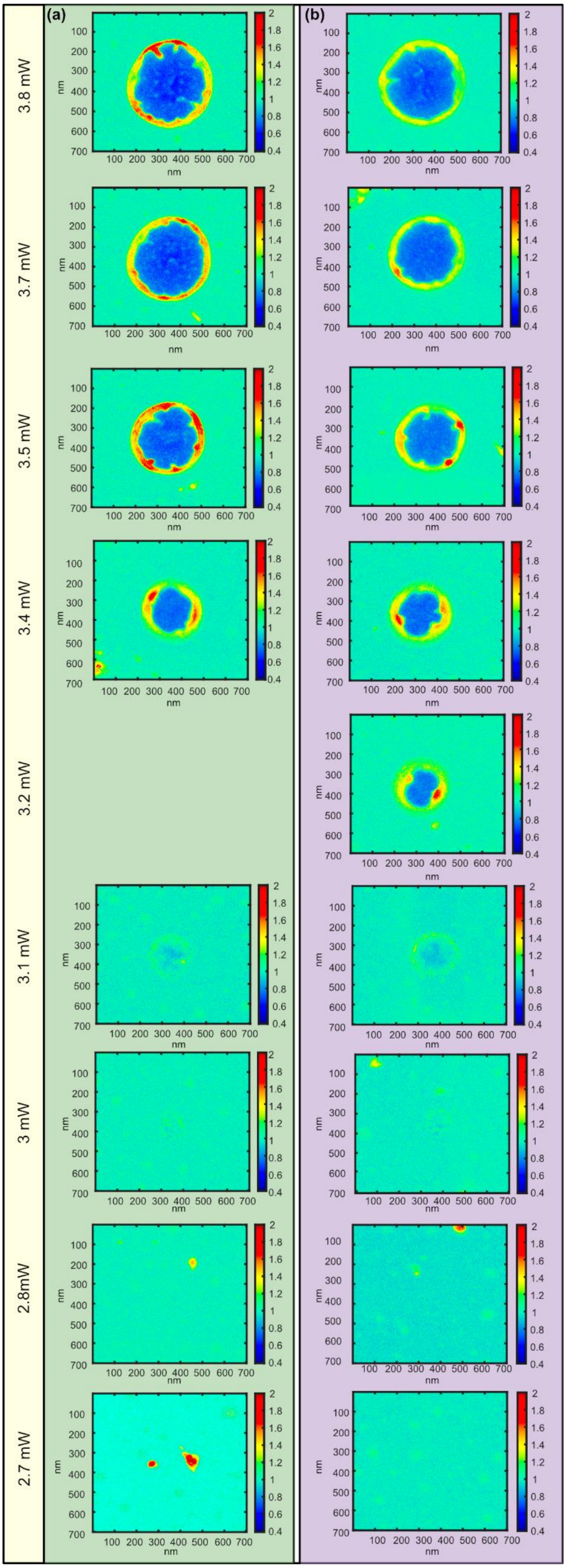
(a)
(b)
3.8 mW
3.7 mW
3.5 mW
3.4 mW
3.2 mW
3.1 mW
3 mW
2.8mW
2.7 mW
nm
100 200 300 400 500 600 700
2
1.8
1.6
1.4
1.2
1
0.8
0.6
0.4

Figure S5. Normalized EFTEM thickness maps at various laser exposure powers in the range 2.7–3.8 mW. All data was acquired from a series of well-separated single pulse laser exposures on the same 40 x 40 µm membrane. The columns (a) and (b) for the same row represent data from two locations separated by 5 µm on the membrane. As described in the main text, the thinned area, as well as the extent of thinning, are functions of the laser power. In both series of results, there is no visible thickness reduction at laser powers below 3 mW. Each EFTEM thickness map is normalized by the thickness of the unexposed area around each site of laser exposure.

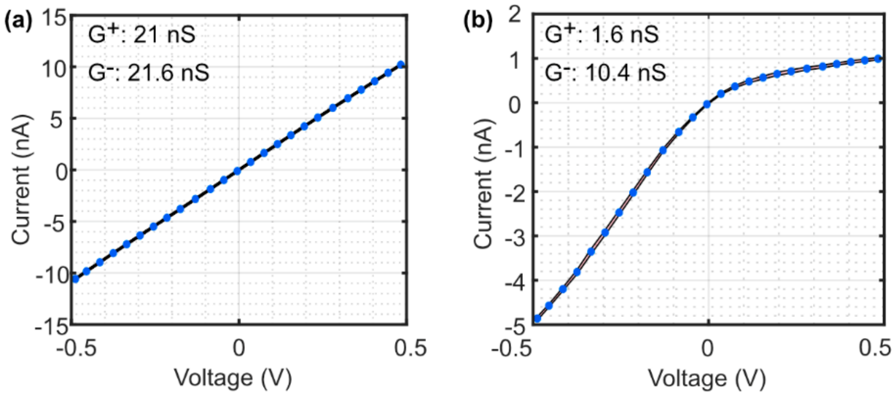

Figure S6. I-V characteristics of nanopores made by pulsed CBD after laser exposure. (a) Eight out of ten nanopores produced by pulsed CBD showed near symmetric and linear I-V characteristics, whereas (b) two nanopores showed current rectification. Such rectification has been previously reported for nanopores fabricated by CBD[2, 3]. In case of significant rectification, where the conductance at positive and negative voltage bias, G^+ and G^- respectively, is significantly different, the nanopore diameter was calculated using the higher conductance value so that the nanopore diameter was not underestimated. The diameter of the rectifying nanopore (b) was estimated to be ~ 8 nm using G^-.

Table S1. Summary of nanopores size change rate per day for 10 samples. The nanopores are fabricated with CBD at different voltage biases. Change rate per day = (d_{final}-d_{init})/ Storage duration[4], where d_{init} represents the nominal nanopore diameter immediately after the fabrication and d_{final} corresponds the nominal nanopore diameter after a certain number of days of storage in 1:1 ethanol:DIW solution. All conductance measurements were performed in 1M KCl. Since we are tracking relative changes in total conductance of the same nanopore, using the nominal nanopore diameter as an intuitive scale, the relatively large uncertainty (~20–40 %) in the nanopore diameter is not significant in this discussion and is therefore not shown.

S. No.	CBD voltage [V]	Laser power [mW]	Nominal pore length [nm]	d_{init} [nm]	d_{final} [nm]	Storage duration [days]	Change rate per day [nm/day]
1	25	3.2	27	19	18	4	-0.3
2	25	2.9	50	21	20	1	-1
3	25	2.9	50	29	32	2	1.5
4	25	3.1	34	24	27	2	1
5	30	3.5	25	25	23	1	-2

Table S2: Eight laser localized CBD nanopores with which DNA translocation experiments were performed. Nanopores where the DNA sensing was successful are marked with an "S" and the nanopores that failed with an "F". Three main modes of failure in observing translocation events were: (1) nanopore got blocked early on during translocation experiments and could not be unblocked; (2) highly unstable nanopore baseline current or (3) featureless current trace with no current spikes after introducing DNA even after several tens of minutes of observation. All nanopores were conditioned in the same way by storing them in 1:1 ethanol:DIW solution for at least one day after CBD. The baseline current noise expressed as I_{rms} at 700 mV at four different bandwidths (100 Hz, 1 kHz, 10 kHz and 100 kHz) for all eight nanopores are also shown.

**Name**	**Laser power (mW)**	**Nanopore diameter (nm)**	**DNA (S/F)**	**I_{rms} [pA] at various bandwidths**			
				**100 Hz**	**1 kHz**	**10 kHz**	**100 kHz**
Pore 1	3.1	18 ± 4	S	266	299	359	535
Pore 2	2.9	20-7	F: no events detected	201	220	251	506
Pore 3	3.1	27 ± 5	F: Pore blocked.	96	111	146	382
Pore 4	2.9	33 -10	S	134	146	172	413
Pore 5	3.1	20 ± 5	F: Pore blocked/ current drifts a lot. No events detected.	113	130	164	337
Pore 6	3.5	23 ± 5	S	343	380	424	522
Pore 7	3.2	26 ± 5	F: No events detected	136	185	238	1520
Pore 8	3.0	27 ± 5	F: No events detected	122	133	150	269

Table S3. Summary of DNA translocation data obtained from three nanopores. The nominal nanopore diameter along with bounds ($d_{nominal}$) calculated as described in Reponse 6, the nanopore diameter from molecular ruler measurements using 2kbp Calf Thymus DNA (d_{MR}), the capture rate and total number of events are all shown.

Name	Laser power [mW]	DNA analyte type	DNA Concentration [μg/mL]	KCl [M]	$d_{nominal}$ [nm]	d_{MR} [nm]	Capture rate [events/min] (Total events)
Pore 1	3.1	λDNA	20	1	18 ± 4	N.A.	61 (1833)
		Calf Thymus	715	1	20 ± 5	12 ± 2	134 (744)
Pore 4	2.9	λDNA	20	1	33 - 10	N.A.	15 (377)
Pore 6	3.5	Calf Thymus	100	1	23 ± 5	16 ± 1	47 (967)

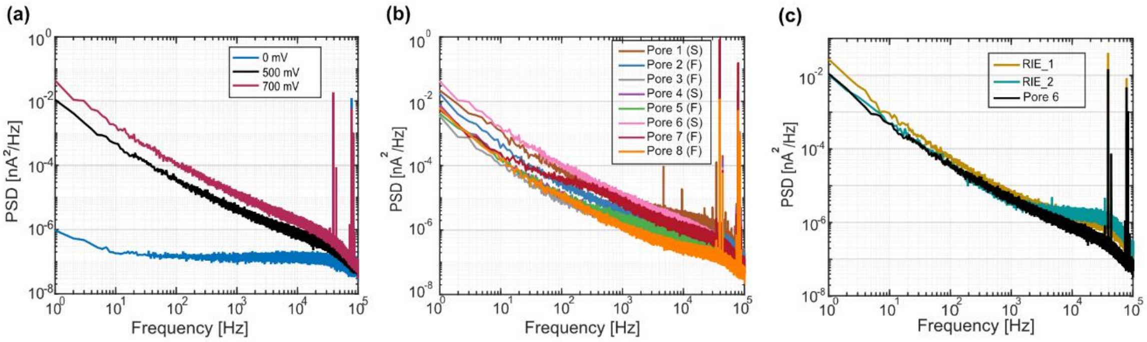

Figure S7. Power spectrum density (PSD) of the ionic current obtained from various nanopores in this study. (a) PSD of the ionic current at various voltage biases for Pore 6 (Table S2). (b) PSD at 700 mV of eight nanopores where DNA translocations were successfully observed. The legend shows the pore names as in Table S2. (c) PSD at 500 mV of the laser localized nanopore in panel a of 23 nm nominal diameter (L~ 25nm) compared to two nanopores of nominal diameter 22 nm and 23 nm produced by CBD on RIE thinned silicon nitride (L~27 nm). The silicon nitride membranes used in all cases originated from the same batch of wafers and was ~50 nm thick at the start.

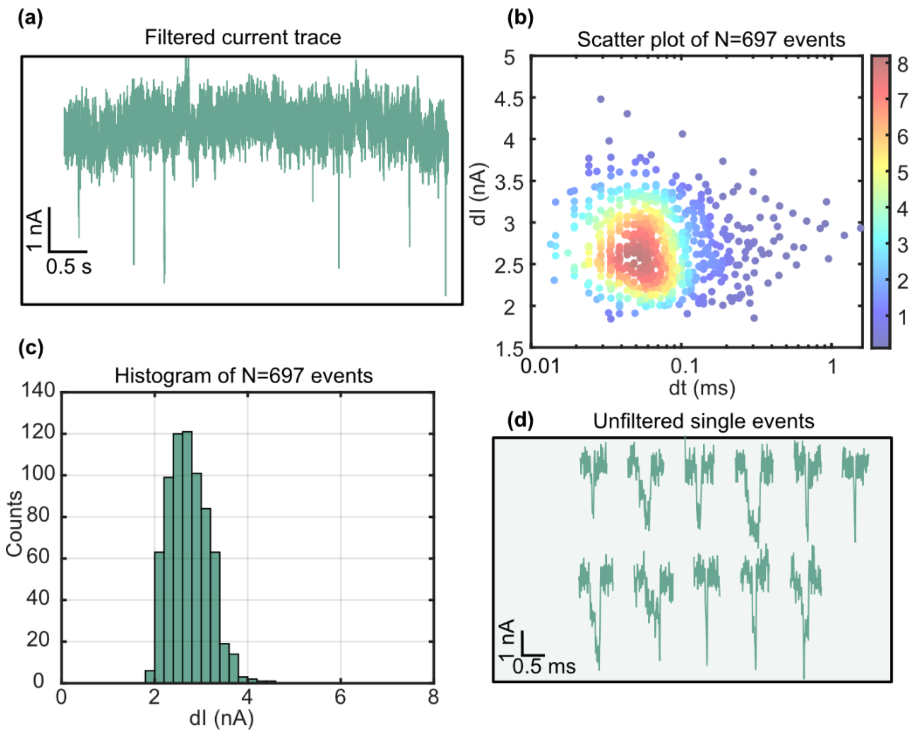

Figure S8. DNA translocation experiments using Pore 6 (see Table S2). (a) Current trace for 20nM 2kbp Calf Thymus dsDNA molecules translocating through a ~ 23 ± 5 nm diameter localized nanopore at a bias voltage of 700 mV (200 kHz sampling rate, low pass filtered to 20 kHz for visualization (b) Heatmap scatter plot of the current blockade (dI) and dwell time (dt) of all 697 events identified at 700 mV. (c) Histogram of current blockade of all events. (d) Selection of extracted events.

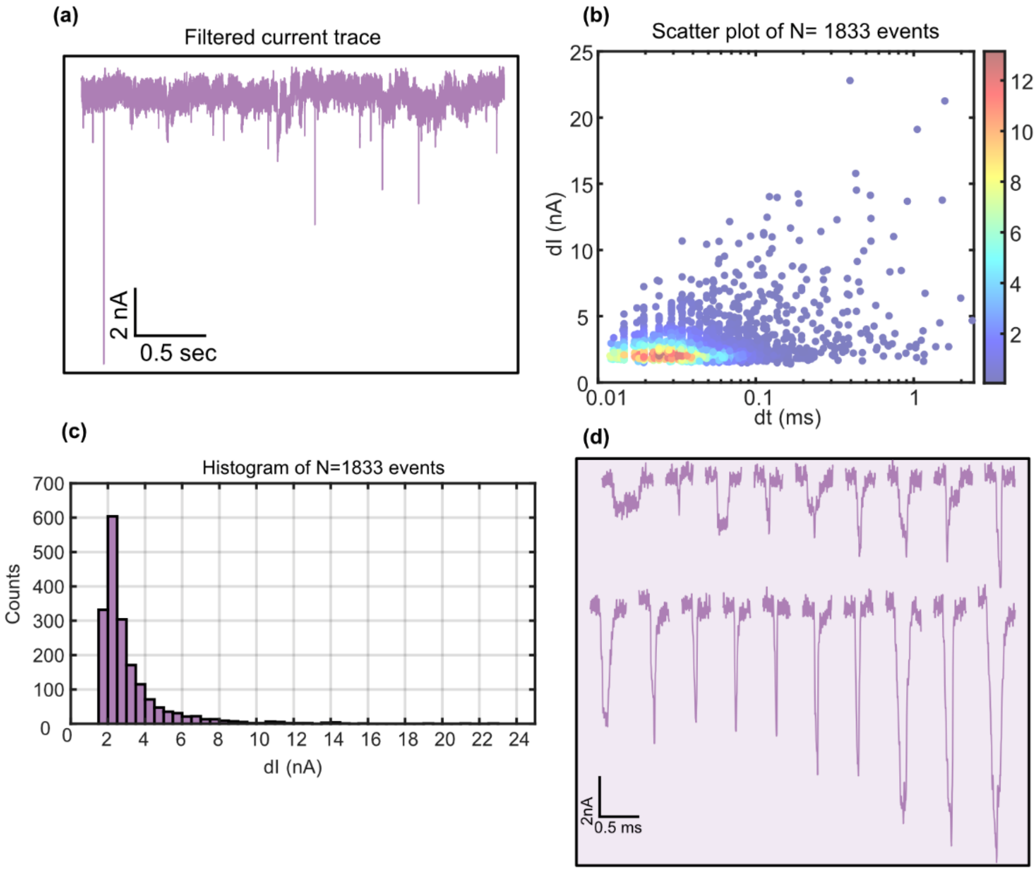

Figure S9. Lambda DNA translocation experiments. (a) Filtered current trace of lambda DNA with final concentration of 20 µg/mL in 1 M KCl. The nanopore size is 18 ± 4 nm and the trace is recorded at 700 mV. The sampling rate is 200 kHz and the data is low-pass filtered to 20 kHz for visualization. (b) Scatter plot of the current blockades versus dwell time with heatmap of 1833 events at 700 mV. (c) Histogram of current blockages of all identified translocation events. (d) Specific events with different current amplitudes corresponding to distinct levels identified in the histogram shown in panel (c).

References

(1) Zrehen, A.; Gilboa, T.; Meller, A. Real-time Visualization and Sub-Diffraction Limit Localization of Nanometer-Scale Pore Formation by Dielectric Breakdown. *Nanoscale* **2017**, *9*, 16437-16445.

(2) Yanagi, I.; Akahori, R.; Takeda, K.-I. Stable Fabrication of a Large Nanopore by Controlled Dielectric Breakdown in a High-pH Solution for the Detection of Various-Sized Molecules. *Scientific Reports* **2019**, *9*.

(3) Chenyu Wen, S. Z., Shiyu Li, Zhen Zhang, Shi-Li Zhang On Rectification of Ionic Current in Nanopores. *Anal.Chem.* **2019**, *91*, 14597−14604.

(4) Chou, Y.-C.; Masih Das, P.; Monos, D. S.; Drndić, M. Lifetime and Stability of Silicon Nitride Nanopores and Nanopore Arrays for Ionic Measurements. *ACS Nano* **2020**, *14* (6), 6715-6728.
